# Lysosome‐Featured Cell Aggregate‐Released Extracellular Vesicles Regulate Iron Homeostasis and Alleviate Post‐Irradiation Endothelial Ferroptosis for Mandibular Regeneration

**DOI:** 10.1002/advs.202505070

**Published:** 2025-06-23

**Authors:** Yuan‐Yuan Li, Bo Ma, Jia‐Wei Lu, Kai‐Chao Zhang, Chao Ma, Sheng‐Feng Bai, Yan‐Jiao Li, Si‐Qi Ying, Wei‐Zong Weng, Kai Zhang, Xi‐Wang Hu, Rang Li, Chen‐Xi Zheng, Xiao‐Ru Xu, Ji Chen, Fang Jin, Hao‐Kun Xu, Jian‐Wei Xie, Yan Jin, Yi Shuai, Bing‐Dong Sui

**Affiliations:** ^1^ State Key Laboratory of Oral and Maxillofacial Reconstruction and Regeneration National Clinical Research Center for Oral Diseases Shaanxi International Joint Research Center for Oral Diseases Center for Tissue Engineering School of Stomatology The Fourth Military Medical University Xi'an Shaanxi 710032 China; ^2^ Department of General Dentistry Xiamen University Affiliated Chenggong Hospital Xiamen Fujian 361001 China; ^3^ State Key Laboratory of Oral & Maxillofacial Reconstruction and Regeneration National Clinical Research Center for Oral Diseases Shaanxi Clinical Research Center for Oral Diseases Department of Orthodontics School of Stomatology The Fourth Military Medical University Xi'an Shaanxi 710032 China; ^4^ Laboratory of Toxicant Analysis Academy of Military Medical Sciences Beijing 100850 China; ^5^ Department of Orthopedics Xiamen University Affiliated Chenggong Hospital Xiamen Fujian 361001 China; ^6^ State Key Laboratory of Oral and Maxillofacial Reconstruction and Regeneration School of Stomatology The Fourth Military Medical University Xi'an Shaanxi 710032 China; ^7^ School of Stomatology Xi'an Medical University Xi'an Shaanxi 710021 China; ^8^ Department of Stomatology Jinling Hospital Affiliated Hospital of Medical School Nanjing University Nanjing Jiangsu 210002 China; ^9^ Department of Stomatology General Hospital of Eastern Theater Command Nanjing Jiangsu 210002 China

**Keywords:** extracellular vesicles, ferroptosis, iron, irradiation, lysosomes, stem cells, tissue regeneration

## Abstract

Ferroptosis, a form of regulated cell death driven by iron accumulation and lipid peroxidation, is implicated in various diseases, but effective therapeutic strategies remain limited. Lysosomal impairments cause iron dysregulation and initiate ferroptosis, which potentially contribute to ionizing radiation‐induced tissue damages. Here, the role of intercellular lysosomal regulation in governing iron homeostasis and protecting against ferroptosis is investigated in models of stem cell aggregation and mandibular regeneration post‐irradiation. Lysosomes are discovered to accumulate in specific regions within multi‐stem cell aggregates and regulate cell aggregate formation based on iron control, which is occurred through hypoxic signaling‐driven lysosomal redistribution mediated by extracellular vesicles. These vesicles exhibit lysosomal features and possess iron‐regulating properties, which rescue lysosomal defects to restore iron homeostasis and mitigate ferroptosis in recipient endothelial cells against the irradiation challenge. Based on lysosomal regulation and anti‐ferroptosis, these cell aggregate‐released extracellular vesicles (CA‐EVs) stimulate the growth of CD31^+^endomucin^+^ specialized vessels despite irradiation both in vitro and in vivo, which further promote bone regeneration of post‐irradiation mandibular defect. These findings highlight the potential of taking CA‐EVs as natural therapeutic agents to safeguard lysosomal function, modulate iron metabolism, and protect against ferroptosis, paving an avenue for combating post‐irradiation endothelial injuries and enhancing tissue regeneration.

## Graphical abstract

Lysosomal redistribution occurs via extracellular vesicles in multi‐stem cell aggregates and controls iron homeostasis for safeguarding cell aggregation. These cell aggregate‐released extracellular vesicles (CA‐EVs) rescue lysosomal impairments and exert anti‐ferroptosis effects in recipient irradiated endothelial cells. Accordingly, CA‐EVs can be harnessed for therapeutic use in promoting CD31^+^endomucin (EMCN)^+^ specialized angiogenesis and promote bone regeneration of the post‐irradiation mandibular defect.

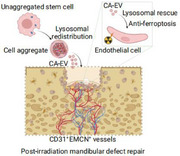

## 1 Introduction

Iron is an essential element in the body. It plays an important role in a variety of fundamental biological and physiological processes, such as oxygen transport, cellular energy generation, extracellular matrix (ECM) synthesis, and tissue homeostasis and regeneration.^[^ ^1^, ^2^, ^3^, ^4^ ^]^ However, excessive iron reacts with oxygen and polyunsaturated fatty acid (PUFA)‐containing lipids to generate membrane lipid peroxides, the accumulation of which results in ferroptosis, a regulated form of cell death.^[^ ^5^, ^6^, ^7^, ^8^ ^]^ Central to the regulation of iron homeostasis are lysosomes, which serve as a nexus incorporating the endocytic iron uptake, the reduction of ferric iron (Fe^3+^) to ferrous iron (Fe^2+^) based on the acidic compartment, the release of Fe^2+^ into the cytosol, and the recycling of iron via the autophagic degradation of ferritin, the iron‐storage protein.^[^ ^8^, ^9^, ^10^ ^]^ Recent studies have further revealed the critical role of lysosomal iron activity in the initiation of ferroptosis, which triggers membrane lipid oxidation in lysosomes, causes lysosomal membrane permeabilization, and subsequently leads to lysosomal iron leakage, fostering cell‐wide lipid peroxidation.^[^ ^11^, ^12^ ^]^ Deprivation of the lysosomal contents, including metal ions within them, is identified as an important cellular anti‐ferroptosis system.^[^ ^13^ ^]^ Notably, lysosomal iron dysregulation and ferroptosis contribute to various tissue damages in pathological states, such as ionizing radiation‐induced epithelial and endothelial injuries.^[^ ^14^, ^15^, ^16^, ^17^ ^]^ However, challenges remain in rescuing lysosomal defects and targeting ferroptosis to promote tissue recovery, with no currently available approach for potential translational and clinical applications.

Lysosomes are integral to regulating not only the endocytic but also the exocytic pathways, the latter of which release cellular contents at least partially dependent on membrane‐encapsulated nanoparticles, recognized as extracellular vesicles (EVs).^[^ ^18^, ^19^, ^20^ ^]^ Our previous research has demonstrated that beyond the secretion of EVs being related to lysosomal regulation, EVs protect lysosomal function in recipient cells based on activation of lysosomal biogenesis.^[^ ^21^, ^22^ ^]^ We have further documented that upon internalization, EVs may directly assemble with membranous organelles in recipient cells and empower the incorporation of the transferred organelle components.^[^ ^23^, ^24^ ^]^ However, whether these vesicles are featured with lysosomal properties and regulate recipient lysosomes against ferroptotic stimuli has not been elucidated. Intriguingly, we and others have reported the enhanced and specialized EV‐mediated intercellular crosstalk in conditions where cells synergize to behave as a collective, such as in stem cell aggregates (CAs), a development‐associated regenerative approach characterized by high‐density stem cells embedded in self‐deposited ECM.^[^ ^24^, ^25^, ^26^, ^27^, ^28^, ^29^ ^]^ Investigating this process further as a potential therapeutic strategy for ferroptosis may offer a promising avenue for future research.

This study aimed to investigate the role of lysosomal regulation by EVs in safeguarding iron homeostasis, with the ultimate goal of developing a therapeutic strategy for counteracting ferroptosis and boosting tissue regeneration. Lysosomal function and iron homeostasis were systematically examined in models of stem cell aggregation and irradiation‐induced endothelial cell (EC) injury. It was hypothesized that cell aggregate‐released extracellular vesicles (CA‐EVs) possess lysosomal properties that regulate iron homeostasis and counteract ferroptosis through lysosomal modulation. This hypothesis was further tested using a murine model of post‐irradiation mandibular bone defect to examine the effects of CA‐EVs on endothelial ferroptosis mitigation in vivo, which potentially promotes CD31^+^endomucin^+^ (CD31^+^EMCN^+^) vessel growth and bone healing. The findings suggest that CA‐EVs represent a promising therapeutic strategy for translational and clinical conditions involving ferroptosis and impaired tissue regeneration.

## 2 Results

### 2.1 Lysosomal Enrichment and Redistribution Occur in Multi‐Stem Cell Aggregates and Regulate CA Formation

**Figure 1:**
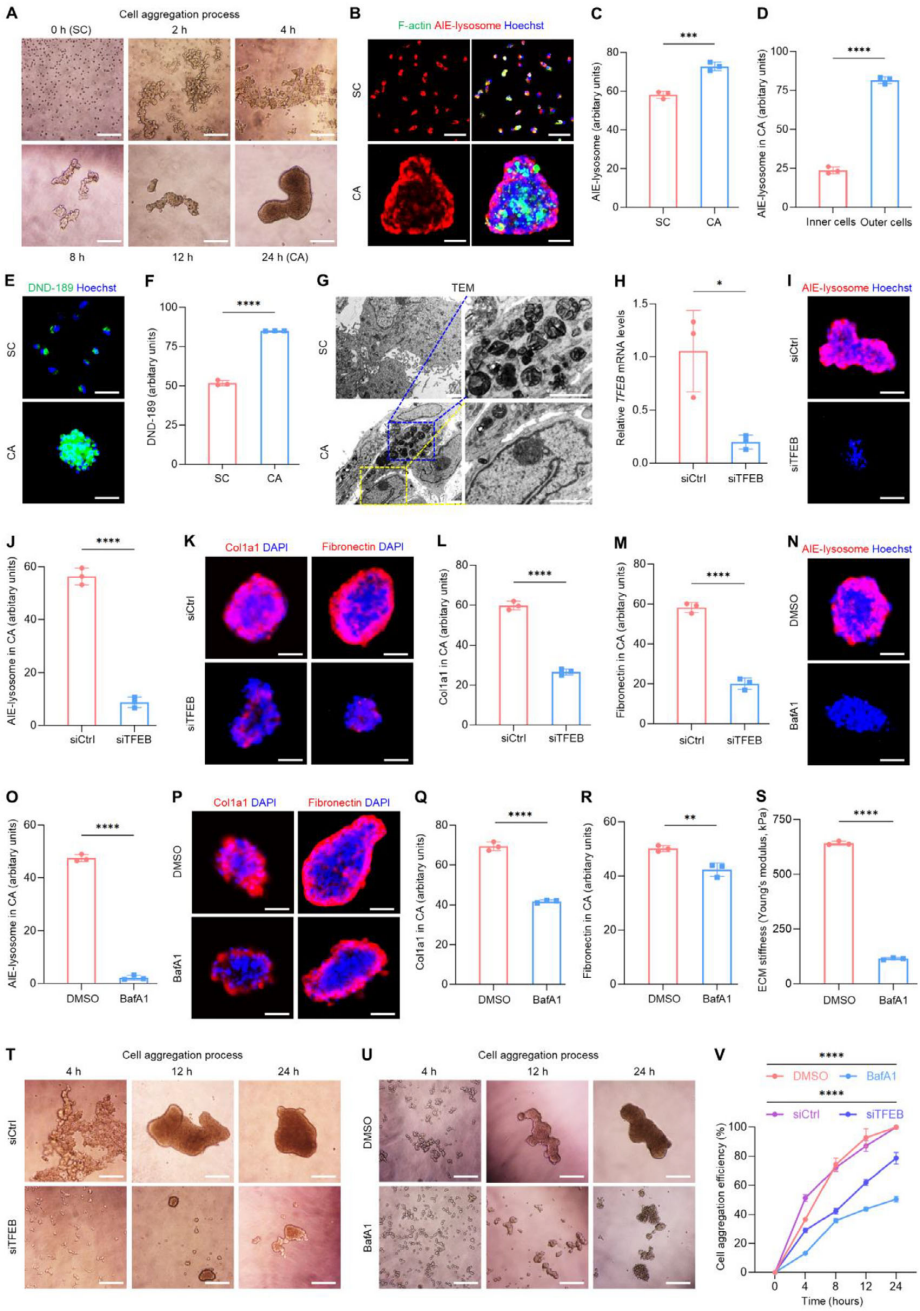
Lysosomal enrichment, redistribution, and function contribute to CA formation. A) Bright‐field images for the process of cell aggregation. SC, unaggregated stem cell; CA, cell aggregate. Scale bars, 150 µm. B) Fluorescent staining of AIE‐lysosome (red) co‐stained with F‐actin (green). Nuclei are stained with Hoechst (blue). Scale bars, 50 µm. C) Quantification of AIE‐lysosome fluorescent intensity in (B). D) Quantification of AIE‐lysosome fluorescent intensity among the outer cells and the inner cells of CAs. E) Fluorescent staining of the pH indicator, lysosensor DND‐189 (green), with nuclei stained with Hoechst (blue). Scale bars, 50 µm. F) Quantification of DND‐189 fluorescent intensity in (E). G) TEM observations. The cell within the blue dashed box indicates a lysosome‐rich cell, while the cell within the yellow dashed box indicates a lysosome‐deficient cell. Scale bars, 2 µm. H) The inhibitory efficacy of siRNAs on *TFEB* expression levels in CAs was evaluated by qRT‐PCR. I) Fluorescent staining of AIE‐lysosome (red) in CAs, with nuclei stained with Hoechst (blue). Scale bars, 50 µm. J) Quantification of AIE‐lysosome fluorescent intensity in (I). (K) Immunostaining for Col1a1 or fibronectin (red) in CAs, with nuclei stained with DAPI (blue). Scale bars, 50 µm. L,M) Quantifications of Col1a1 and fibronectin fluorescent intensity in (K). siCtrl, siRNA negative control; siTFEB, siRNA oligonucleotides of *transcription factor EB*. N) Fluorescent staining of AIE‐lysosome (red) in CAs, with nuclei stained with Hoechst (blue). Scale bars, 50 µm. O) Quantification of AIE‐lysosome fluorescent intensity in (N). P) Immunostaining for Col1a1 or fibronectin (red) in CAs, with nuclei stained with DAPI (blue). Scale bars, 50 µm. Q,R) Quantifications of Col1a1 and fibronectin fluorescent intensity in (P). S) ECM stiffness of CA analyzed by the AFM nanoindentation test. DMSO, dimethyl sulfoxide; BafA1, bafilomycin A1, a lysosomal V‐ATPase inhibitor. T,U) Bright‐field images for the process of cell aggregation. Scale bars, 150 µm. V) Quantification of cell aggregation efficiency. Results are expressed as mean ± SD. *n* = 3 samples per group for each experimental readout. *p* values were calculated using Student's *t*‐test (C,D,F,H,J,L,M,O,Q,R,S) or two‐way ANOVA with Šídák's multiple comparisons test V). ^*^ *p* < 0.05, ^**^ *p* < 0.01, ^***^ *p* < 0.001, and ^****^ *p* < 0.0001.

To begin, we investigated the role of lysosomes in regulating intercellular dynamics using an established model of stem cell aggregation, in which single stem cells (SCs) were cultured in ultra‐low attachment (ULA) plates to induce the formation of CAs.^[^ ^27^ ^]^ The CAs typically formed within 24 h (**Figure** **1**A). Different types of connections between the aggregated stem cells and between cells and the ECM, were observed in CAs (Figure S1A–H, Supporting Information). Lysosomes in CAs and unaggregated SCs were visualized using an aggregation‐induced emission (AIE)‐lysosome probe. A larger number of lysosomes were detected in CAs compared to unaggregated SCs (Figure 1B,C). Notably, the outer cells of CAs contained more lysosomes than the inner cells (Figure 1D). Furthermore, lysosomal acidity was evaluated using the fluorescent pH indicator, lysosensor green DND‐189, which fluoresces in acidic environments. The pH in CAs was found to be lower than that in unaggregated SCs, indicating the presence of more acidic lysosomes in CAs (Figure 1E,F). Transmission electron microscopy (TEM) further confirmed a significant increase in lysosome number in CAs compared to unaggregated SCs. Interestingly, lysosome distribution within CAs was heterogeneous, with some cells displaying a high lysosome content and others showing only a small number of lysosomes (Figure 1G). These findings demonstrate that the aggregation of SCs into CAs enhances lysosome abundance and causes lysosomal redistribution, highlighting the potential role of lysosomes in regulating intercellular dynamics.

Next, we investigated whether lysosomes are indeed required for the formation of CAs. *Transcription factor EB* (*TFEB*), the master gene for lysosomal biogenesis,^[^ ^30^ ^]^ was selected as the target for small interfering RNA (siRNA)‐based knockdown of gene expression. Quantitative real‐time polymerase chain reaction (qRT‐PCR) analysis confirmed the gene knockdown efficiency of siRNA oligonucleotides of *TFEB* (siTFEB) in CAs (Figure 1H). Expectedly, compared to CAs transfected with the siRNA negative control (siCtrl), CAs after siTFEB transfection showed a reduction in lysosomal abundance (Figure 1I,J). Moreover, protein levels of α‐1 type I collagen (Col1a1) and fibronectin, major components of ECM in CAs,^[^ ^27^ ^]^ were suppressed after siTFEB transfection, correlated with smaller CAs formed (Figure 1K–M). Notably, the mRNA levels of ECM genes, including *COL1A1*, *FN1* (encoding fibronectin), *LAMC1* (encoding laminin), and *DCN* (encoding decorin), remained paralleled in CAs despite siTFEB transfection, suggesting unaffected gene transcription (Figure S2A–D, Supporting Information). The above results highlighted the necessity of lysosomal presence to CA formation. With regard to lysosomal function, the acidic environment of lysosomes is maintained by vacuolar H⁺‐adenosine triphosphatase (V‐ATPase) and other ion channels.^[^ ^31^ ^]^ Accordingly, the impact of lysosomal acidification on CA formation was assessed by adding bafilomycin A1 (BafA1; a V‐ATPase inhibitor) to the culture medium. Treatment with BafA1 decreased lysosome content in CAs (Figure 1N,O). Furthermore, treatment with BafA1 reduced the levels of both Col1a1 and fibronectin in CAs without influencing the ECM gene transcription (Figure 1P–R; Figure S2E–H, Supporting Information). The functional assay of ECM stiffness measured by atomic force microscopy (AFM) nanoindentation experiments provided crucial evidence to link lysosomal acidification to biomechanical ECM changes, demonstrating diminished mechanical properties of the CA‐ECM after BafA1 treatment (Figure 1S). Importantly, either siTFEB transfection or BafA1 treatment slowed CA formation (Figure 1T–V). Collectively, these findings suggest that lysosomes play a critical role in regulating the establishment of multi‐stem cell aggregates.

### 2.2 Hypoxic Response Drives Lysosomal Dynamics in CAs for Critical Regulation of Iron Homeostasis

**Figure 2:**
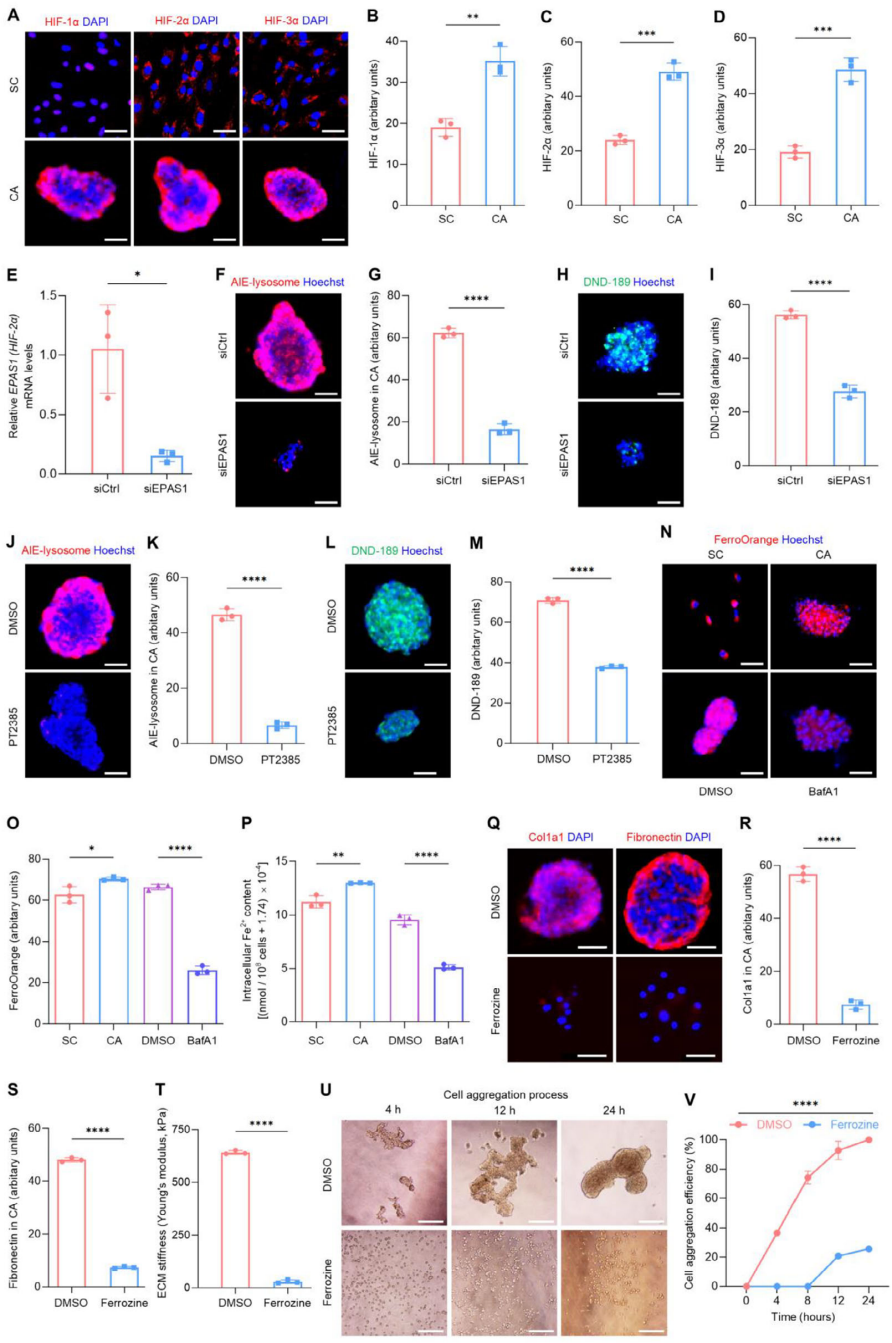
Lysosomal dynamics is driven by HIF‐2α signaling and regulates iron homeostasis in CAs. A) Immunostaining for HIF‐1α, HIF‐2α, or HIF‐3α (red), with nuclei stained with DAPI (blue). Scale bars, 50 µm. B–D) Quantifications of HIF‐1α, HIF‐2α, and HIF‐3α fluorescent intensity in (A). SC, unaggregated stem cell; CA, cell aggregate. E) The inhibitory efficacy of siRNAs on *EPAS1* (encoding HIF‐2α) expression levels in CAs was evaluated by qRT‐PCR. F) Fluorescent staining of AIE‐lysosome (red) in CAs, with nuclei stained with Hoechst (blue). Scale bars, 50 µm. G) Quantification of AIE‐lysosome fluorescent intensity in (F). H) Fluorescent staining of the pH indicator, lysosensor DND‐189 (green) in CAs, with nuclei stained with Hoechst (blue). Scale bars, 50 µm. I) Quantification of DND‐189 fluorescent intensity in (H). siCtrl, siRNA negative control; siEPAS1, siRNA oligonucleotides of *endothelial PAS domain‐containing protein 1* (*EPAS1*). (J) Fluorescent staining of AIE‐lysosome (red), with nuclei stained with Hoechst (blue). Scale bars, 50 µm. K) Quantification of AIE‐lysosome fluorescent intensity in (J). L) Fluorescent staining of DND‐189 (green) in CAs, with nuclei stained with Hoechst (blue). Scale bars, 50 µm. M) Quantification of DND‐189 fluorescent intensity in (L). DMSO, dimethyl sulfoxide; PT2385, a HIF‐2α inhibitor. N) Fluorescent staining of the ferrous iron (Fe^2+^) indicator, FerroOrange (red), with nuclei stained with Hoechst (blue). Scale bars, 50 µm. O) Quantification of FerroOrange fluorescent intensity in (N). P) Intracellular Fe^2+^ content was analyzed by a colorimetric method. BafA1, bafilomycin A1, a lysosomal V‐ATPase inhibitor. Q) Immunostaining for Col1a1 or fibronectin (red), with nuclei stained with DAPI (blue). Scale bars, 50 µm. R,S) Quantifications of Col1a1 and fibronectin fluorescent intensity in (Q). T) ECM stiffness of CAs analyzed by the AFM nanoindentation test. U) Bright‐field images for the process of cell aggregation. Scale bars, 150 µm. V) Quantification of cell aggregation efficiency. Ferrozine, a Fe^2^⁺ chelator. Results are expressed as mean ± SD. *n* = 3 samples per group for each experimental readout. *p* values were calculated using Student's *t*‐test (B,C,D,E,G,I,K,M,R,S,T), one‐way ANOVA with Tukey's post hoc test O,P), or two‐way ANOVA (V). ^*^ *p* < 0.05, ^**^ *p* < 0.01, ^***^ *p* < 0.001, and ^****^ *p* < 0.0001.

We then investigated why lysosomal changes occurred in CAs and how lysosomes were functionally required for CA formation. Reduced oxygen availability during cell aggregation results in the activation of hypoxia‐inducible factors (HIFs), a family of transcription factors that regulate cellular responses to low oxygen levels.^[^ ^32^, ^33^ ^]^ Accordingly, we examined the expression of HIFs and discovered that the three key molecules, HIF‐1α, HIF‐2α, and HIF‐3α, were all upregulated in CAs, compared to unaggregated SCs (**Figure** **2**A–D). Particularly, HIF‐2α is reported to long persist and stabilize in the stem cell aggregation.^[^ ^32^ ^]^ To focus on whether HIF‐2α contributes to lysosomal regulation during cell aggregation, siRNA‐based knockdown of *endothelial PAS domain‐containing protein 1* (*EPAS1*), the gene encoding HIF‐2α, was performed (Figure 2E). The results showed that transfection of siRNA oligonucleotides of *EPAS1* (siEPAS1) caused a decline of both the lysosomal content and the lysosomal acidity in CAs (Figure 2F–I). To further examine the impact of HIF‐2α on lysosomes in CAs, cells were treated with PT2385, a specific HIF‐2α inhibitor, during CA formation. Also, chemical inhibition of HIF‐2α resulted in a reduction in both lysosomal content and lysosomal acidification in CAs (Figure 2J–M). These findings suggest that HIF‐2α contributes to lysosome regulation in CA formation.

Previous studies have documented that the iron ion, specifically Fe^2+^, catalyzes the ECM protein synthesis by serving as a cofactor of prolyl and lysyl hydroxylases.^[^ ^34^, ^35^ ^]^ Furthermore, the lysosomal function is critical for the regulation of iron homeostasis.^[^ ^9^ ^]^ Enlightened by aformentioned findings that lysosomes contributed to ECM deposition of CAs without affecting the ECM gene transcription (Figure 1K,L,P–S; Figure S2A–H, Supporting Information), we continued to decipher that whether Fe^2+^ was the key downstream mediator of lysosomal control of CA formation. The Fe^2+^‐specific fluorescent probe, FerroOrange, was adopted. Interestinly, the Fe^2^⁺ level was significantly higher in CAs compared to unaggregated SCs. After treatment with BafA1, the Fe^2^⁺ concentration in CAs was remarkably reduced, indicating the vital importance of lysosomal function in maintaining iron homeostasis in CAs (Figure 2N,O). Further examination of intracellular Fe^2^⁺ content by a colorimetric complementary method confirmed the fluorescent findings, demonstrating increased Fe^2^⁺ content in CAs governed by lysosomal acidification (Figure 2P). Then, to assess the impact of intracellular Fe^2^⁺ on CA formation, ferrozine, a Fe^2^⁺ chelator, was introduced into the culture medium. The inclusion of ferrozine reduced Col1a1 and fibronectin levels in CAs without changing the ECM gene transcription (Figure 2Q–S; Figure S2I–L, Supporting Information). Moreover, ferrozine treatment diminished the ECM stiffness of CAs and also slowed the CA formation (Figure 2T–V). Together, these findings highlight the critical role of lysosome‐controlled intracellular Fe^2^⁺ in the regulation of cell aggregation.

### 2.3 CA‐EVs Possess Lysosomal Features and Mediate Intercellular Lysosomal Redistribution

**Figure 3:**
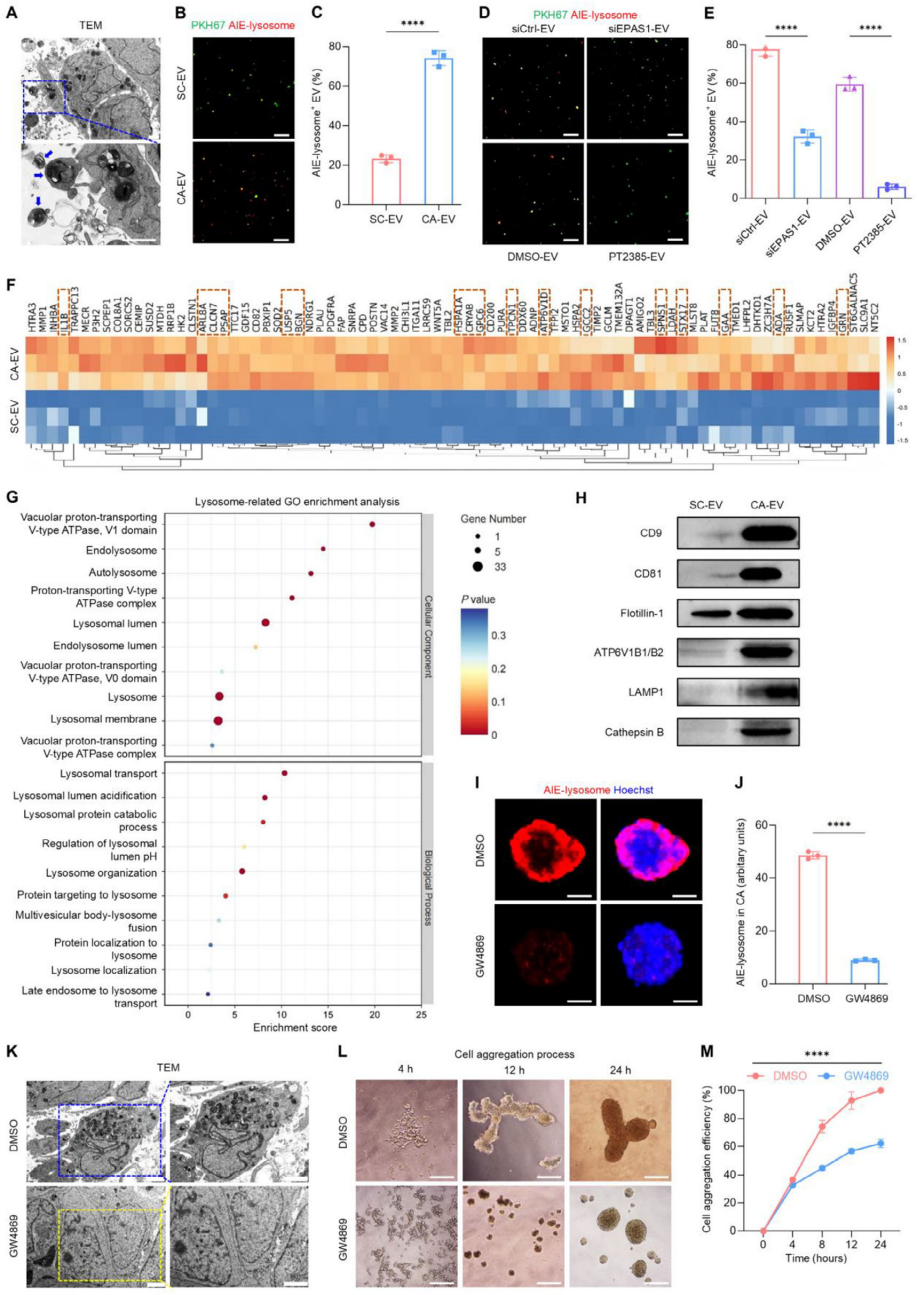
CA‐EVs share lysosomal features and mediate lysosomal redistribution. A) TEM observations of CAs. The extracellular vesicles (EVs) within the blue dashed box contain lysosomes. Scale bars, 2 µm. B) Fluorescent staining of AIE‐lysosome (red) co‐stained with PKH67 (green) in EVs. Scale bars, 10 µm. C) Quantification of the proportion of AIE‐lysosome^+^ EVs in (B). SC‐EV, extracellular vesicle secreted by an unaggregated stem cell; CA‐EV, cell aggregate‐released extracellular vesicle. D) Fluorescent staining of AIE‐lysosome (red) co‐stained with PKH67 (green) in CA‐EVs. Scale bars, 10 µm. E) Quantification of the proportion of AIE‐lysosome^+^ EVs in (D). siCtrl‐EV, EV secreted by CA treated with siRNA negative control; siEPAS1‐EV, EV secreted by CA treated with siRNA oligonucleotides of *endothelial PAS domain‐containing protein 1* (*EPAS1*, encoding HIF‐2α); DMSO‐EV, EV secreted by CA treated with dimethyl sulfoxide; PT2385‐EV, EV secreted by CA treated with PT2385, a HIF‐2α inhibitor. F) The top proteins that are significantly upregulated in CA‐EVs compared to SC‐EVs, with protein abundance normalized using Z‐scores. Rows correspond to individual proteins, while columns represent separate replicates. Proteins within the orange dashed boxes indicate lysosome‐associated proteins. G) Gene Ontology (GO) enrichment analysis of upregulated differentially expressed proteins (DEPs) in CA‐EVs compared to SC‐EVs. The *Y*‐axis represents lysosome‐related GO terms, while the *X*‐axis shows the enrichment score. The color of the bubbles indicates the significance of enrichment. The size of the bubbles reflects the number of proteins associated with each GO term. H) Protein expression levels in EVs. I) Fluorescent staining of AIE‐lysosome (red), with nuclei stained with Hoechst (blue). Scale bars, 50 µm. J) Quantification of AIE‐lysosome fluorescent intensity in (I). K) TEM observations of CAs. CAs treated with DMSO are enriched with lysosomes and ECM components (within the blue dashed box), while CAs treated with GW4869 lack lysosomes and ECM components (within the yellow dashed box). Scale bars, 2 µm. L) Bright‐field images for the process of cell aggregation. Scale bar, 150 µm. M) Quantification of cell aggregation efficiency. GW4869, an inhibitor of EV release. Results are expressed as mean ± SD. *n* = 3 samples per group for each experimental readout. *p* values were calculated using Student's *t*‐test (C,J), one‐way ANOVA with Tukey's post hoc test (E), or two‐way ANOVA (M). ^****^ *p* < 0.0001.

During the research above, we intriguingly discovered that the distribution of lysosomes altered in CAs, with being more highly enriched in the outer cells of CAs compared to the inner cells (Figure 1B,D). We then checked closely the TEM images to examine if there was a possible reason for the appearance of this unusual lysosomal distribution pattern. Surprisingly, ultrastructural examination identified numerous EVs in the ECM of CAs, some of which contained lysosomes (**Figure** **3**A). The CA‐EVs were subsequently isolated and characterized using a previously published protocol.^[^ ^36^ ^]^ When examined with TEM, the CA‐EVs displayed a characteristic spherical morphology with a bilayer membrane structure (Figure S3A, Supporting Information). Nanoparticle tracking analysis (NTA) showed that the diameters of the CA‐EVs ranged from 50 to 1000 nm, with a peak distribution between 95 and 125 nm (Figure S3B, Supporting Information). Western blots revealed that the CA‐EVs were enriched in marker proteins such as CD9, CD81, and Flotillin‐1, while exhibiting low levels of Golgin‐84 (Figure S3C, Supporting Information). To examine the potential lysosomal content in CA‐EVs, the vesicles were labeled with PKH67 and analyzed using the AIE‐lysosome probe. The lysosome‐positive rate of CA‐EVs was higher than that EVs secreted by unaggregated SCs (SC‐EVs; Figure 2B,C), accompanied by increased yield of EVs in cell aggregation (Figure S4A, Supporting Information). Notably, both the release of EVs and the encapsulation of lysosomes within EVs were dependent on the HIF‐2α signaling in CAs, because that siEPAS1 transfection suppressed the production and lysosome‐positive rate of CA‐EVs (Figure 3D,E; Figure S4A, Supporting Information). Also, treating CAs with PT2385, the specific HIF‐2α inhibitor, reduced the lysosome‐positive rate of EVs secreted (Figure 3D,E). Interestingly, however, hypoxia preconditioning of CAs by 1% O_2_ did not alter the lysosomal content (Figure S4B,C, Supporting Information), lysosomal acidity (Figure S4D,E, Supporting Information), or EV secretion (Figure S4F, Supporting Information). These results indicated that a sufficient hypoxic microenvironment was established in CAs, thus extra hypoxia had limited additional beneficial effects on CAs and CA‐EVs. Moreover, PT2385 treatment directly on collected CA‐EVs did not alter their lysosomal content (Figure S4G,H, Supporting Information) or affect the vesicle number (Figure S4I, Supporting Information), suggesting that hypoxic regulation mainly occured during CA formation and EV secretion by CA per se.

Protein extracts derived from CA‐EVs and SC‐EVs were then subjected to liquid chromatography‐tandem mass spectrometry (LC‐MS/MS) for analysis of the proteome (Table S1, Supporting Information). The investigation identified 229 proteins that were significantly upregulated in CA‐EVs compared to SC‐EVs, including multiple lysosome‐associated proteins (Figure 3F). Gene ontology (GO) enrichment analysis highlighted several lysosome‐related terms in the upregulated differentially expressed proteins (DEPs) in CA‐EVs, including those associated with lysosomal acidification, such as “vacuolar proton‐transporting V‐type ATPase, V1 domain,” “proton‐transporting V‐type ATPase complex,” and “regulation of lysosomal lumen pH” (Figure 3G). Consistent with these findings, lysosome acidity‐related proteins, such as ATP6V1D (a component of the V‐ATPase complex), were upregulated in CA‐EVs in the proteomic data (Figure 3F). Western blots further confirmed increased levels of EV‐specific proteins (CD9, CD81, Flotillin‐1) and lysosome‐associated proteins (ATP6V1B1/B2, lysosomal‐associated membrane protein 1 [LAMP1], Cathepsin B) in CA‐EVs, compared to SC‐EVs (Figure 3H). These results indicated that CA‐EVs possess lysosomal features. To evaluate the impact of EV release on intercellular lysosomal redistribution, as well as CA formation, GW4869, an inhibitor of EV release, was added to the culture medium. Expectedly, this treatment suppressed lysosomes during CA formation, as proved by fluorescent probing (Figure 3I,J) and TEM analysis (Figure 3K). Moreover, GW4869 treatment delayed the CA formation process (Figure 3L,M). These findings suggest that CA‐EVs play a critical role in safeguarding the formation of multi‐stem cell aggregates.

### 2.4 CA‐EVs Rescue Lysosomal Function and Promote Angiogenesis of ECs Despite the Irradiation Challenge

**Figure 4:**
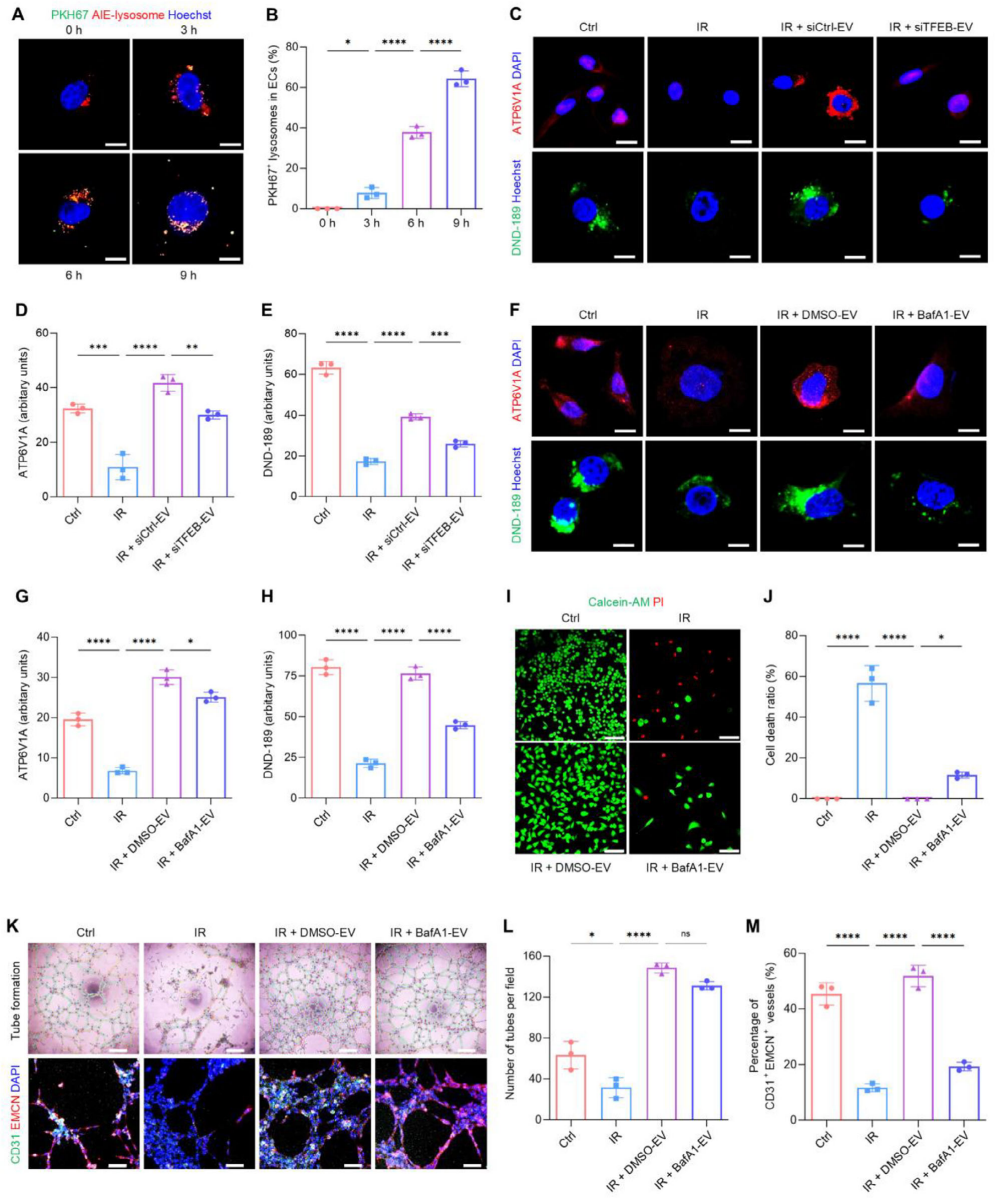
CA‐EVs rescue lysosomal function and promote angiogenesis of irradiated ECs. A) CA‐EVs (stained with PKH67, green) uptake by HUVECs (stained with AIE‐lysosome, red), with nuclei stained with Hoechst (blue). Scale bars, 10 µm. B) Quantification of the proportion of PKH67^+^ lysosomes (merged with internalized CA‐EVs) in HUVECs. C) Immunostaining for ATP6V1A (red) with nuclei stained with DAPI (blue), or fluorescent staining of the pH indicator, lysosensor DND‐189 (green), with nuclei stained with Hoechst (blue) in HUVECs. Scale bars, 10 µm. D,E) Quantifications of ATP6V1A and DND‐189 fluorescent intensity in (C). Ctrl, control; IR, irradiation; siCtrl‐EV, EV secreted by CA treated with siRNA negative control; siTFEB‐EV, EV secreted by CA treated with siRNA oligonucleotides of *transcription factor EB*. F) Immunostaining for ATP6V1A (red) with nuclei stained with DAPI (blue), or fluorescent staining of DND‐189 (green) with nuclei stained with Hoechst (blue) in HUVECs. Scale bars, 10 µm. G,H) Quantifications of ATP6V1A and DND‐189 fluorescent intensity in (F). I) Live (Calcein‐AM, green) and dead (PI, red) HUVECs measured by cell viability/cytotoxicity assay. Scale bars, 100 µm. J) Quantification of cell death ratio in (I). K) Bright‐field images of the tube formation assay of HUVECs, and immunostaining for CD31 (green) with endomucin (EMCN, red) of these tubes, with nuclei stained with DAPI (blue). Scale bars, 400 µm (up) and 100 µm (down). L) Quantification of formed tubes in (K). M) Quantification for the proportion of CD31^+^EMCN^+^ vessels relative to total vessels in (K). DMSO‐EV, CA‐EV treated with dimethyl sulfoxide; BafA1‐EV, CA‐EV treated with bafilomycin A1. Results are expressed as mean ± SD. *n* = 3 samples per group for each experimental readout. *p* values were calculated using one‐way ANOVA with Tukey's post hoc test. ^*^ *p* < 0.05, ^**^ *p* < 0.01, ^***^ *p* < 0.001, and ^****^ *p* < 0.0001; ns, not significant (*p* > 0.05).

Next, the potential of CA‐EVs in modulating cells with lysosomal defects was investigated. Recent studies have reported that lysosomal impairments contribute to ionizing radiation‐induced tissue damages, which involve EC injury and dysfunction.^[^ ^17^, ^37^ ^]^ Accordingly, PKH26‐labeled CA‐EVs were introduced to cultured human umbilical vein endothelial cells (HUVECs), and their internalization was confirmed through confocal laser scanning microscopy (CLSM) (**Figure** **4**A,B). The impact of CA‐EVs on the lysosomal function of irradiated HUVECs was then examined. Exposure of HUVECs to 8 Gy X‐ray resulted in a decrease in the expression of ATP6V1A (a component of the lysosomal V‐ATPase complex), and a reduction in the number of acidic lysosomes (Figure 4C–H). Treatment with CA‐EVs restored ATP6V1A expression and increased the quantity of acidic lysosomes in the irradiated cells (Figure 4C–H). To further clarify the role of lysosomal components and related functions mediating the effects of CA‐EVs, the vesicles were collected from siTFEB‐transfected CAs or pretreated with BafA1 prior to application to ECs. Either deprivation of lysosomes or inhibition of lysosomal function in CA‐EVs led to a decline of the expression of ATP6V1A and the number of acidic lysosomes in irradiated ECs when compared with the respective experimental control (Figure 4C–H). Further research by live/dead staining confirmed that CA‐EVs ameliorated the cell death of HUVECs provoked by irradiation, which was also dependent on the lysosomal V‐ATPase activity of CA‐EVs (Figure 4I,J). Moreover, the tube formation assay showed that irradiated ECs formed significantly fewer tubes than non‐irradiated controls (Figure 4K,L). Immunofluorescence staining of these tubes revealed a reduction in CD31 and EMCN co‐expression (Figure 4K,M), indicating diminished formation of CD31^+^EMCN^+^ vessels, the specialized capillary subtype critical for supporting tissue homeostasis.^[^ ^38^ ^]^ Treatment with CA‐EVs improved the angiogenic capacity in irradiated ECs with an increased proportion of CD31^+^EMCN^+^ vessels relative to total vessels (Figure 4K–M). In contrast, pretreating CA‐EVs with BafA1 reduced the formation of CD31^+^EMCN^+^ vessels compared to the control CA‐EVs (Figure 4K,M). Together, these findings demonstrate that lysosome‐featured CA‐EVs enhance lysosomal function in recipient cells with lysosomal defects, which protect ECs from irradiation‐induced injury and promote angiogenesis.

### 2.5 CA‐EVs Restore Iron Homeostasis Through Lysosomal Regulation in Irradiated ECs

**Figure 5:**
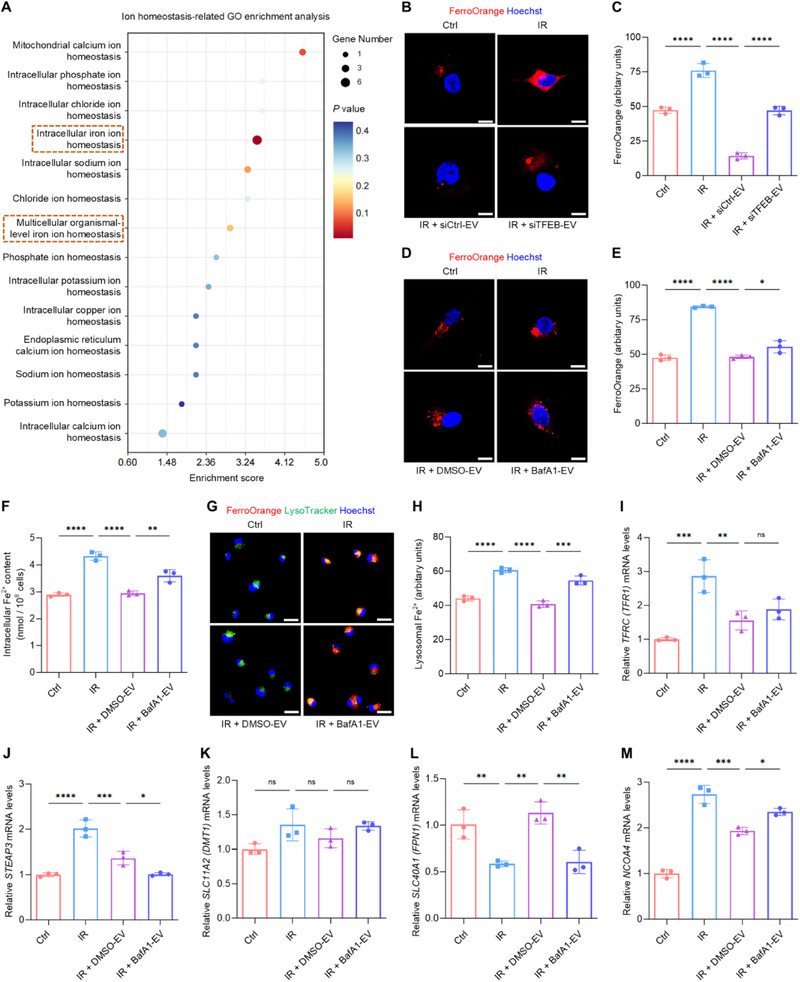
CA‐EVs restore iron homeostasis against the irradiation challenge in ECs through lysosomal regulation. A) Gene Ontology (GO) enrichment analysis of upregulated differentially expressed proteins (DEPs) in CA‐EVs compared to SC‐EVs. The *Y*‐axis represents ion homeostasis‐related GO terms, while the *X*‐axis shows the enrichment score. The color of the bubbles indicates the significance of enrichment. The size of the bubbles reflects the number of proteins associated with each GO term. Terms within the orange dashed boxes indicate iron ion‐associated terms. B) Fluorescent staining of the ferrous iron (Fe^2+^) indicator, FerroOrange (red), with nuclei stained with Hoechst (blue). Scale bars, 50 µm. C) Quantification of FerroOrange fluorescent intensity in (B). Ctrl, control; IR, irradiation; siCtrl‐EV, EV secreted by CA treated with siRNA negative control; siTFEB‐EV, EV secreted by CA treated with siRNA oligonucleotides of *transcription factor EB*. D) Fluorescent staining of FerroOrange (red) with nuclei stained with Hoechst (blue). Scale bars, 50 µm. E) Quantification of FerroOrange fluorescent intensity in (D). F) Intracellular Fe^2+^ content in HUVECs analyzed by a colorimetric method. G) Fluorescent staining of FerroOrange (red) co‐stained with LysoTracker (green), with nuclei stained with Hoechst (blue). Scale bars, 30 µm. H) Quantification of lysosomal Fe^2+^ fluorescent intensity in (G). I–M) Gene expression levels of *TFRC* (encoding *TFR1*), *STEAP3*, *SLC11A2* (encoding *DMT1*), *SLC40A1* (encoding *FPN1*), and *NCOA4* in HUVECs were determined by qRT‐PCR. DMSO‐EV, CA‐EV treated with dimethyl sulfoxide; BafA1‐EV, CA‐EV treated with bafilomycin A1. Results are expressed as mean ± SD. *n* = 3 samples per group for each experimental readout. *p* values were calculated using one‐way ANOVA with Tukey's post hoc test. ^*^ *p* < 0.05, ^**^ *p* < 0.01, ^***^ *p* < 0.001, and ^****^ *p* < 0.0001; ns, not significant (*p* > 0.05).

We continued to investigate whether CA‐EVs were capable of modulating iron homeostasis of recipient cells via lysosomal regulation. GO enrichment analysis was again used to in‐depth understand the role of CA‐EVs in ion homeostasis by comparing CA‐EVs to SC‐EVs. Among the ion homeostasis‐related terms, only “intracellular iron ion homeostasis” showed significant enrichment (**Figure** **5**A). FerroOrange staining demonstrated that irradiation significantly elevated intracellular Fe^2^⁺ concentrations in ECs, which were effectively reduced by CA‐EVs (Figure 5B–E). These effects of CA‐EVs were suppressed when the vesicles were collected from siTFEB‐transfected CAs or pretreated with BafA1 prior to application to ECs (Figure 5B–E), indicating the indispensable role of lysosomal regulation. Colorimetric analysis of Fe^2^⁺ content in ECs post‐irradiation confirmed the beneficial effects of CA‐EVs in controlling iron homeostasis based on lysosomal regulation (Figure 5F). Furthermore, specific probing of lysosomal Fe^2^⁺ by co‐staining of FerroOrange and LysoTracker exhibited that irradiation caused lysosomal iron dysregulation, which was alleviated by CA‐EV treatment dependent on the lysosomal V‐ATPase activity (Figure 5G,H). Additional experiments were performed to analyze the iron metabolic routes in ECs.^[^ ^8^ ^]^ Expression of transferrin receptor 1 (TFR1, the mediator of cellular iron uptake through endocytosis, encoded by *TFRC*), six‐transmembrane epithelial antigen of the prostate 3 (STEAP3, a ferrireductase reducing Fe^3+^ to Fe^2+^ in lysosomes), ferroportin 1 (FPN1, the cellular iron exporter, encoded by *SLC40A1*), and nuclear receptor coactivator 4 (NCOA4, a selective cargo receptor mediating the autophagic degradation of ferritin in lysosomes) were revealed to have altered mRNA levels post‐irradiation, whereas divalent metal transporter 1 (DMT1, the major iron exporter in lysosomes, encoded by *SLC11A2*), mitoferrin 2 (MFRN2, a mitochondrial iron transporter, encoded by *SLC25A28*), and iron storage molecules mitochondrial ferritin (FTMT) and ferritin (encoded by *ferritin heavy chain 1*, *FTH1*, and *ferritin light chain*, *FTL*), remained comparable in gene transcription (Figure 5I–M; Figure S5A–D, Supporting Information). These data highlighted that lysosomal iron input was prominently affected by irradiation, corresponding with the accumulated lysosomal Fe^2^⁺ shown by FerroOrange and LysoTracker co‐staining. Notably, after CA‐EV treatment, the altered mRNA expression levels of *TFR1*, *STEAP3*, *FPN1*, and *NCOA4* were all rescued, among which the restoration of *FPN1* and *NCOA4* gene transcription was particularly dependent on lysosomal regulation (Figure 5I–M). Collectively, the above results suggest that CA‐EVs restore iron homeostasis through lysosomal regulation in irradiated ECs.

### 2.6 CA‐EVs Mitigate Ferroptosis Against Irradiation Injury in ECs Through Lysosomal Regulation

**Figure 6:**
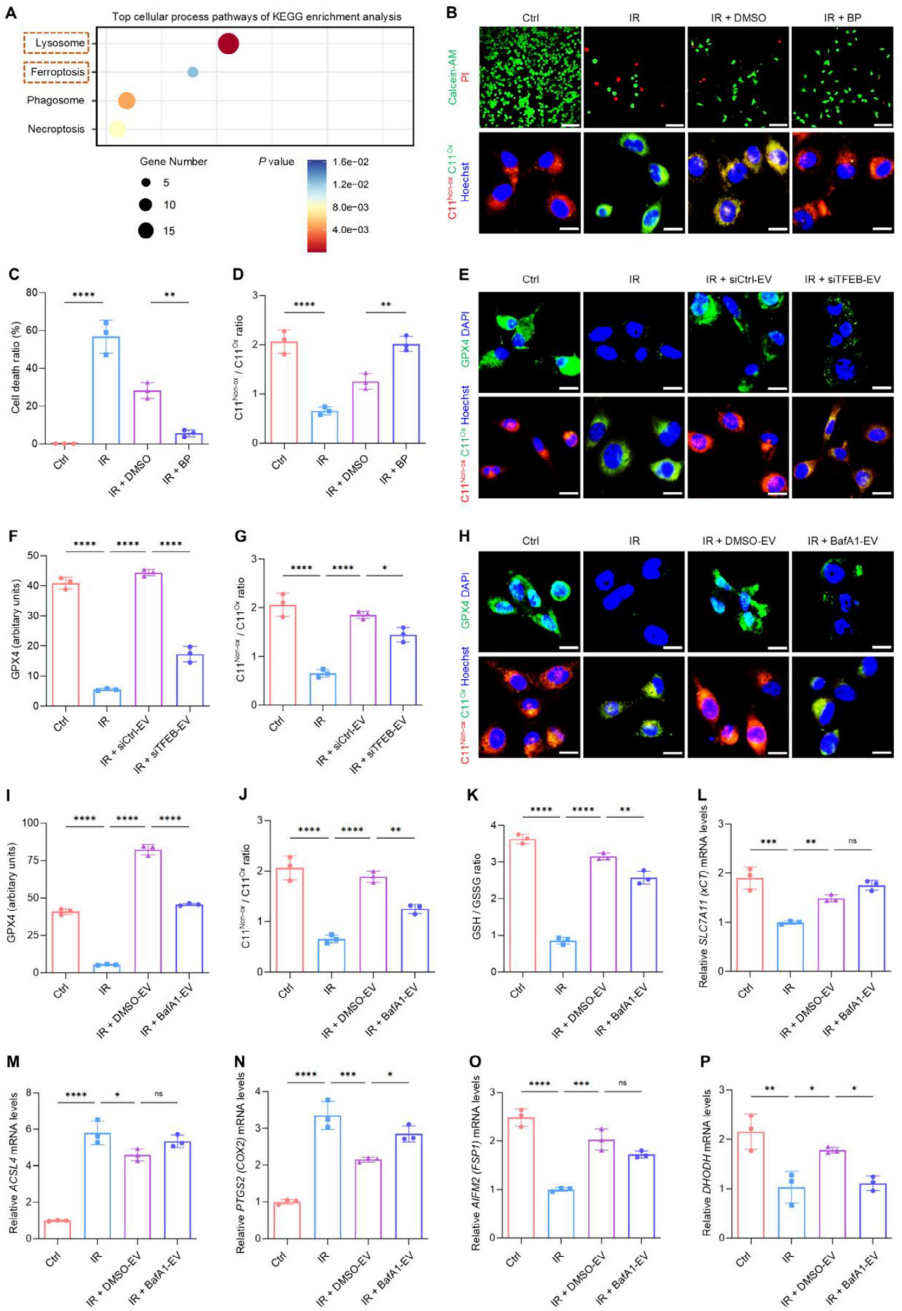
CA‐EVs mitigate ferroptosis against irradiation injury in ECs through lysosomal regulation. A) Kyoto Encyclopedia of Genes and Genomes (KEGG) enrichment analysis of upregulated differentially expressed proteins (DEPs) in CA‐EVs compared to SC‐EVs. The *Y*‐axis represents KEGG terms of cellular processes, while the *X*‐axis shows the enrichment score. The color of the bubbles indicates the significance of enrichment. The size of the bubbles reflects the number of proteins associated with each KEGG term. Terms within the orange dashed boxes indicate lysosome and ferroptosis‐associated terms. B) Fluorescent staining of live (Calcein‐AM, green) and dead (PI, red) HUVECs measured by cell viability/cytotoxicity assay, or C11‐BODIPY (red, non‐oxidized; green, oxidized) in HUVECs with nuclei stained with Hoechst (blue). Scale bars, 100 µm (up) and 10 µm (down). C,D) Quantifications of cell death ratio and non‐oxidized over oxidized C11‐BODIPY ratio in (B). Ctrl, control; IR, irradiation; DMSO, dimethyl sulfoxide; BP, 2,2’‐bipyridyl, a ferrous iron (Fe^2+^) chelator. E) Immunostaining for glutathione peroxidase 4 (GPX4) (green) with nuclei stained with DAPI (blue), or fluorescent staining of C11‐BODIPY (red, non‐oxidized; green, oxidized) with nuclei stained with Hoechst (blue) in HUVECs. Scale bars, 10 µm. F,G) Quantifications of GPX4 fluorescent intensity and non‐oxidized over oxidized C11‐BODIPY ratio in (E). siCtrl‐EV, EV secreted by CA treated with siRNA negative control; siTFEB‐EV, EV secreted by CA treated with siRNA oligonucleotides of *transcription factor EB*. H) Immunostaining for GPX4 (green) with nuclei stained with DAPI (blue), or fluorescent staining of C11‐BODIPY (red, non‐oxidized; green, oxidized) with nuclei stained with Hoechst (blue) in HUVECs. Scale bars, 10 µm. I,J) Quantifications of GPX4 fluorescent intensity and non‐oxidized over oxidized C11‐BODIPY ratio in (H). K) The glutathione (GSH)/glutathione disulfide (GSSG) ratio in HUVECs. (L‐P) Gene expression levels of *SLC7A11* (encoding *xCT*), *ACSL4*, *PTGS2* (encoding *COX2*), *AIFM2* (encoding *FSP1*), and *DHODH* in HUVECs determined by qRT‐PCR. DMSO‐EV, CA‐EV treated with dimethyl sulfoxide; BafA1‐EV, CA‐EV treated with bafilomycin A1. Results are expressed as mean ± SD. *n* = 3 samples per group for each experimental readout. *p* values were calculated using one‐way ANOVA with Tukey's post hoc test. ^*^ *p* < 0.05, ^**^ *p* < 0.01, ^***^ *p* < 0.001, and ^****^ *p* < 0.0001; ns, not significant (*p* > 0.05).

Previous literature has demonstrated that ionizing radiation increases reactive oxygen species (ROS) generation and causes cell death via ferroptosis.^[^ ^14^, ^39^ ^]^ Recent studies have also documented that lysosomal iron dysregulation and lysosomal lipid peroxidation initiate ferroptosis.^[^ ^11^, ^12^ ^]^ In light of these findings, the potential functions of CA‐EVs were further examined through Kyoto Encyclopedia of Genes and Genomes (KEGG) enrichment analysis, by comparing CA‐EVs to SC‐EVs. The analysis identified lysosome and ferroptosis pathways among the top 20 pathways of enrichment (**Figure** **6**A). Indeed, X‐ray irradiation‐triggered cell death in HUVECs was characterized by reduced non‐oxidized/oxidized lipid ratio probed using C11‐BODIPY 581/591 (Figure 6B–D), indicating lipid perioxiation, a hallmark of ferroptosis.^[^ ^40^ ^]^ Furthermore, 2,2′‐bipyridyl (BP), another Fe^2^⁺ chelator, was added to the culture medium of irradiated ECs, which suppressed oxidized lipid levels and ameliorated cell death compared to the control (Figure 6B–D), validating the requirement of iron in the process. Irradiation exposure also significantly reduced glutathione peroxidase 4 (GPX4) levels in HUVECs (Figure 6E,F), which is an essential ferroptosis‐controlling enzyme.^[^ ^8^, ^41^ ^]^ Importantly, treatment with CA‐EVs restored GPX4 expression and alleviated lipid peroxidation in irradiated ECs, indicating anti‐ferroptosis effects (Figure 6E–I). These effects were diminished when using EVs produced by siTFEB‐transfected CAs or pretreated with BafA1 prior to application to ECs (Figure 6E–I). Several other ferroptosis regulators were also investigated, including evaluation of the glutathione (GSH)/glutathione disulfide (GSSG) ratio and analyses of expression levels of the xCT system (the substrate specificity‐conferring subunit of system x_c_^−^ encoded by *SLC7A11*) importing cystine in exchange for glutamate, the pro‐ferroptosis players, acyl‐coenzyme A (CoA) synthetase long‐chain family (ACSL4) and prostaglandin‐endoperoxide synthase 2 (PTGS2, *aka*. cyclooxygenase 2, COX2), and the anti‐ferroptosis components, ferroptosis suppressor protein 1 (FSP1, *aka*. apoptosis‐inducing factor mitochondria‐associated 2, AIFM2) and dihydroorotate dehydrogenase (DHODH) (Figure 6K–P). Intriguingly, all these regulators were altered by irradiation in ECs and were rescued by CA‐EV treatment (Figure 6K–P). In particular, the GSH abundance and gene transcription of *PTGS2* and *DHODH* were modulated by CA‐EVs based on lysosomal regulation (Figure 6K,N,P). Collectively, these findings indicate that CA‐EVs mitigate irradiation‐induced ferroptosis in ECs via a lysosome‐dependent mechanism.

### 2.7 CA‐EVs Exert In Vivo Therapeutic Effects to Combat Irradiated Endothelial Ferroptosis and Promote Specialized Angiogenesis Based on Lysosomal Regulation

**Figure 7:**
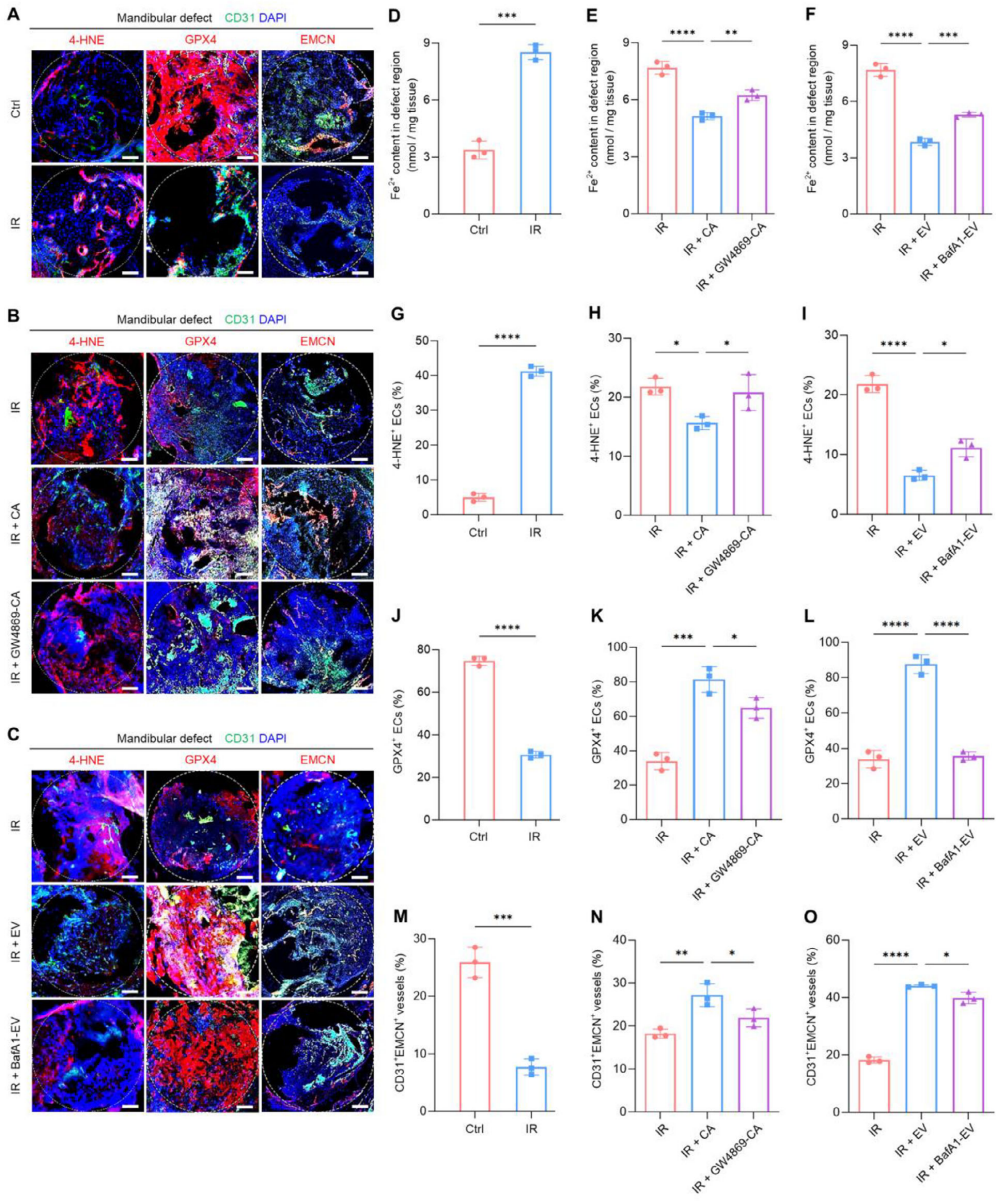
CA‐EVs suppress endothelial ferroptosis and promote the growth of CD31^+^EMCN^+^ vessels in irradiated mandibular defects through lysosomal regulation. A–C) Immunostaining of 4‐hydroxy‐2‐nonenal (4‐HNE), glutathione peroxidase 4 (GPX4), or endomucin (EMCN) (red), co‐stained with CD31 (green), with nuclei stained with DAPI (blue). The white dashed circles indicate the defect area. Scale bars, 100 µm. D–F) Ferrous iron (Fe^2+^) content in the defect region was analyzed by a colorimetric method. G–I) Quantification for the proportion of 4‐HNE^+^ ECs relative to total CD31^+^ ECs in (A–C). J–L) Quantification for the proportion of GPX4^+^ ECs relative to total CD31^+^ ECs in (A–C). M–O) Quantification for the proportion of CD31^+^EMCN^+^ vessels relative to total vessels in (A–C). Ctrl, control; IR, irradiation; CA, cell aggregate; GW4869‐CA, GW4869‐pretreated cell aggregate; EV, extracellular vesicle released from CA; BafA1‐EV, bafilomycin A1‐treated extracellular vesicle. Results are expressed as mean ± SD. *n* = 3 samples per group for each experimental readout. *p* values were calculated using Student's *t*‐test (D,G,J,M), or one‐way ANOVA with Tukey's post hoc test (E,F,H,I,K,L,N,O). ^*^ *p* < 0.05, ^**^ *p* < 0.01, ^***^ *p* < 0.001, and ^****^ *p* < 0.0001.

A post‐irradiation mandibular defect model in mice was further established to investigate the therapeutic effects of CA‐EVs on ferroptosis and angiogenic function of ECs in vivo. Expectedly, 4‐hydroxy‐2‐nonenal (4‐HNE), a critical indicator of lipid peroxidation in ferroptosis,^[^ ^40^ ^]^ dramatically increased in ECs after the irradiation challenge, with elevated Fe^2+^ content in the mandibular defect regions (**Figure** **7**A,D,G). In contrast, endothelial GPX4 levels were decreased upon irradiation (Figure 7A,J), collectively suggesting irradiation‐induced ferroptosis in vivo. Furthermore, the post‐irradiation defects exhibited a reduced proportion of CD31^+^EMCN^+^ vessels relative to total vessels, compared to non‐irradiated defects (Figure 7A,M), indicating a damaged capacity of specialized angiogenesis. CA implantation, notably, significantly mitigated endothelial ferroptosis, reduced the Fe^2+^ content, and increased the proportion of CD31^+^EMCN^+^ vessels in the defect region (Figure 7B,E,H,K,N). CAs were then pretreated before implantation with GW4869, an inhibitor of EV secretion, to determine whether these effects were mediated by CA‐EVs. As a result, application of GW4869‐pretreated CAs to the irradiated defect diminished the capability of CAs to protect ECs against ferroptosis and promote formation of CD31^+^EMCN^+^ vessels (Figure 7B,E,H,K,N). The therapeutic effects of CA‐EVs were directly evaluated by applying PKH26‐labeled CA‐EVs to the defect site, with internalization confirmed using CLSM (Figure S6, Supporting Information). Quantitative analysis indicated that CA‐EV treatment significantly mitigated endothelial ferroptosis, reduced the Fe^2+^ content, and enhanced the proportion of CD31^+^EMCN^+^ vessels in the irradiated defect region (Figure 7C,F,I,L,O). The necessity of lysosomal activity in CA‐EVs was subsequently examined by pretreating the vesicles with BafA1 prior to application, which blocked the therapeutic effects of CA‐EVs (Figure 7C,F,I,L,O). Together, these findings highlight the ability of CA‐EVs to mitigate endothelial ferroptosis and enhance specialized angiogenesis post‐irradiation in vivo, with lysosomal regulation playing a critical role in their therapeutic potential.

### 2.8 CA‐EVs are Effective in Enhancing Bone Regeneration of Post‐Irradiation Mandibular Defect Through Lysosomal Regulation

**Figure 8:**
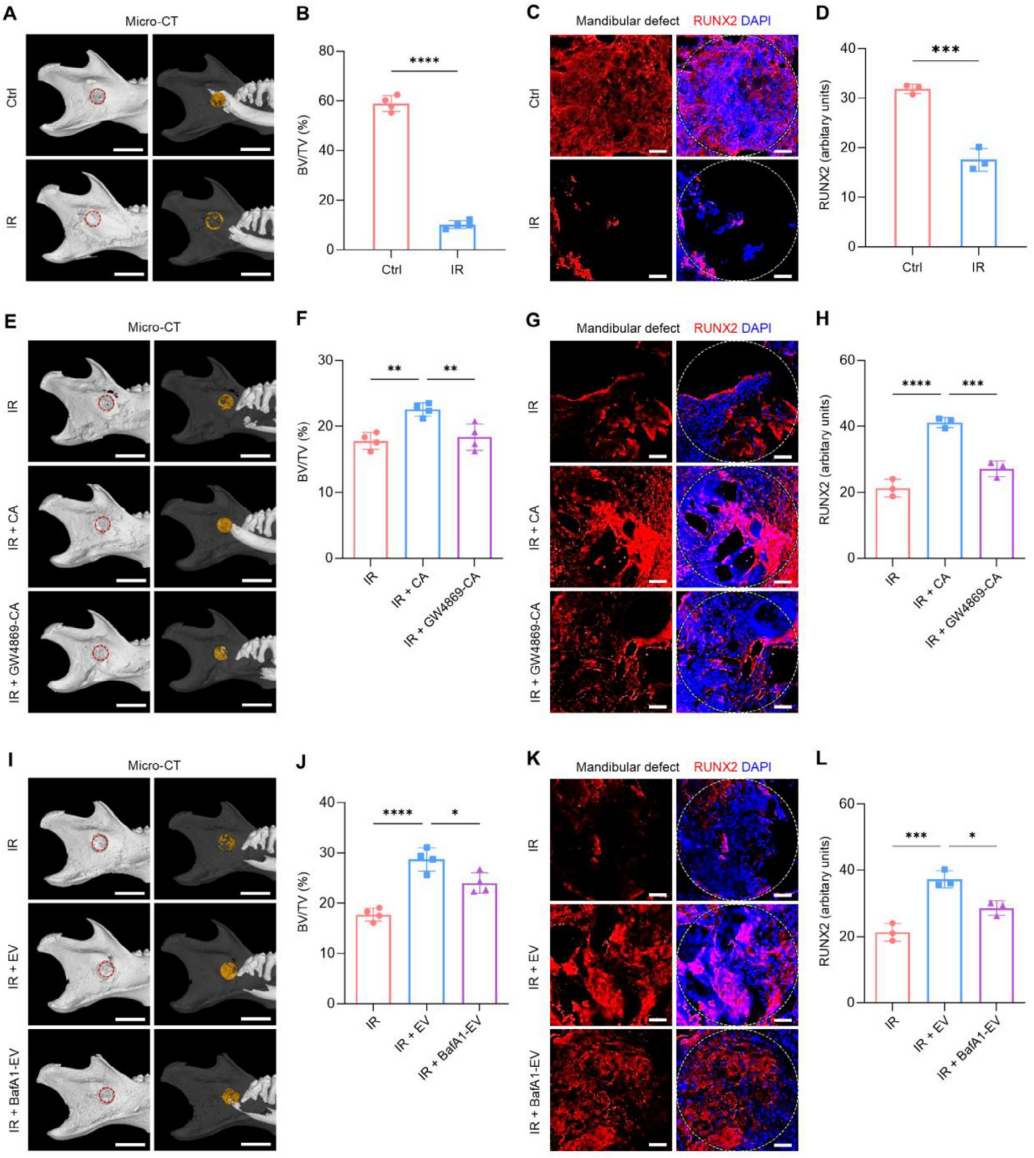
CA‐EVs enhance bone regeneration of post‐irradiation mandibular defect through lysosomal regulation. (A,E,I) Representative 3D images of micro‐CT of mandibles. The red dashed circles indicate the defect areas. The yellow shaded regions represent the new bones. Scale bars, 2 mm. (B,F,J) Quantitative analysis bone volume over tissue volume (BV/TV) in mandibular defect regions. (C,G,K) Immunostaining of Runt‐related Transcription Factor 2 (RUNX2, red) with nuclei stained with DAPI (blue) in mandibular defect regions. The white dashed circles indicate the defect areas. Scale bars, 100 µm. (D,H,L) Quantification of RUNX2 fluorescent intensity in mandibular defect regions. Ctrl, control; IR, irradiation; CA, cell aggregate; GW4869‐CA, GW4869‐pretreated cell aggregate; EV, extracellular vesicle released from CA; BafA1‐EV, bafilomycin A1‐treated extracellular vesicle. Results are expressed as mean ± SD. *n* ≥ 3 samples per group for each experimental readout. *p* values were calculated using Student's *t*‐test (B,D), or one‐way ANOVA with Tukey's post hoc test (F,H,J,L). ^*^ *p* < 0.05, ^**^ *p* < 0.01, ^***^ *p* < 0.001, and ^****^ *p* < 0.0001.

**Figure 9:**
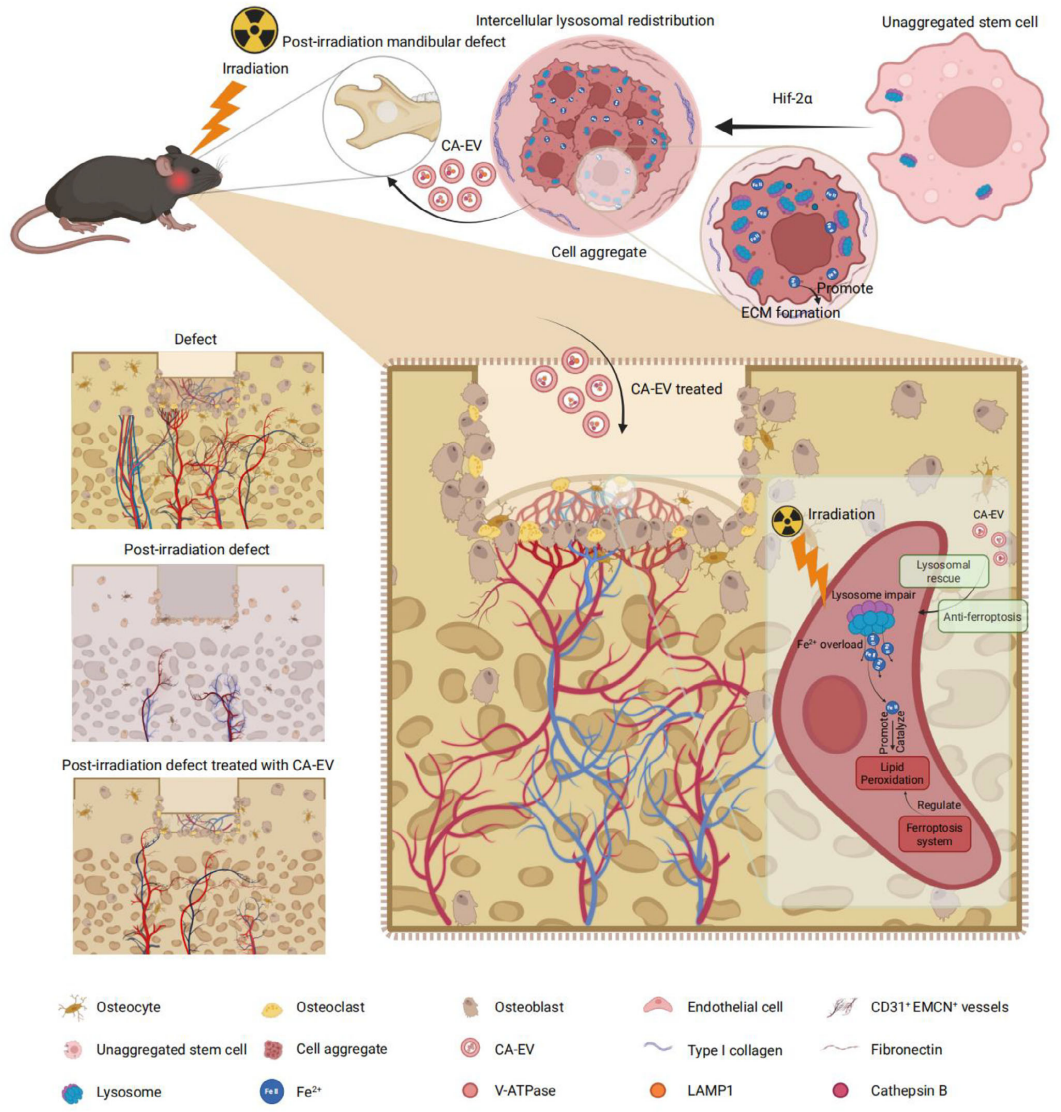
Graphical summary of this study. Lysosomal redistribution occurs via HIF‐2α regulation and is mediated by EVs in multi‐stem cell aggregates, which controls iron homeostasis for promoting ECM synthesis and safeguarding cell aggregation. These CA‐EVs exert anti‐ferroptosis effects in recipient irradiated ECs through the rescue of lysosomal impairments and restoration of iron homeostasis. Accordingly, CA‐EVs can be harnessed for therapeutic use in promoting CD31^+^EMCN^+^ specialized angiogenesis and promoting bone regeneration of the post‐irradiation mandibular defect. CA‐EV, cell aggregate‐released extracellular vesicle; ECM, extracellular matrix; EMCN, endomucin; HIF‐2α, hypoxia‐inducible factors‐2α; LAMP1, lysosomal‐associated membrane protein 1. Figure was created using BioRender (https://www.biorender.com/) in compliance with its Terms of Use.

Formation of CD31^+^EMCN^+^ vessels help establish a pro‐regenerative microenvironment.^[^ ^25^, ^38^ ^]^ Accordingly, micro‐computed tomography (micro‐CT) was used to finally investigate the therapeutic effects of CA‐EVs on promoting post‐irradiation bone repair in vivo. Four weeks after surgery, the bone volume over tissue volume (BV/TV) ratio in post‐irradiation defects was significantly lower compared to non‐irradiated mandibular defects (**Figure** **8**A,B). Immunofluorescence staining of healing bone defects supported these findings, showing diminished expression of runt‐related transcription factor 2 (RUNX2), a master transcription factor for osteoblast differentiation, in post‐irradiation defect areas (Figure 8C,D). Implantation with CAs, nevertheless, significantly improved the BV/TV ratio in the repair of irradiated bone defects, which was suppressed by the pretreatment of CAs with GW4869 (Figure 8E,F). Quantitative analysis of RUNX2 expression in the post‐irradiation defect regions confirmed these findings, suggesting promoted osteogenesis by CA implantation dependent on the EV release (Figure 8G,H). The effects of CA‐EVs were further assessed in the defect region by direct application. Quantitative analysis demonstrated that CA‐EV application significantly increased both the BV/TV value and the RUNX2 expression in the post‐irradiation mandibular defect region compared to the control group, which were abrogated by pretreatment with BafA1 prior to application (Figure 8I–L). Taken together, these results demonstrate that CA‐EVs effectively promote regeneration of irradiated challenging bone defects, with lysosomal regulation playing a critical role in their therapeutic efficacy (**Figure** **9**).

## 3 Discussion

Cell aggregation is a process in which individual cells come together to form clusters or aggregates enriched with self‐deposited ECM.^[^ ^27^, ^42^ ^]^ This process is influenced by multiple factors, such as cell adhesion molecules, chemical signals, or physical forces, which are essential for tissue formation, wound healing, and embryonic development.^[^ ^27^, ^43^ ^]^ Iron plays an important role in maintaining the structural integrity of the ECM.^[^ ^1^, ^4^ ^]^ This essential metal ion interacts with proteins including collagen and fibronectin to form a stable network that is crucial for tissue elasticity and strength.^[^ ^44^ ^]^ In addition, iron acts as a signaling molecule within the ECM. Iron‐dependent enzymes modulate the composition and functionality of the ECM through redox reactions, influencing cellular growth and differentiation.^[^ ^4^ ^]^ The exact role of iron in cell aggregation is not fully understood. Here, intracellular Fe^2^⁺ was found to be essential for cell aggregation and ECM formation in CAs, with lysosomes playing a critical role in regulating intracellular iron homeostasis. Furthermore, inhibiting HIF‐2α disrupts lysosomal activity during cell aggregation. These findings provide a foundation for further research into the role of iron homeostasis in cell aggregation.

The biogenesis and release of EVs are regulated by the interplay between endosomal and lysosomal systems. This interplay highlights their critical roles in cellular communication and waste management.^[^ ^45^, ^46^, ^47^ ^]^ Lysosomes influence EV release by degrading intraluminal vesicles within multivesicular bodies (MVB). When the MVB fuses with lysosomes, the intraluminal vesicles are degraded to prevent their release.^[^ ^48^, ^49^ ^]^ This mechanism regulates the quantity and quality of EVs secreted by cells. Conversely, EVs may be taken up by recipient cells via different endocytic pathways. Once internalized, EVs may release their cargo into the cytoplasm or be directed to lysosomes for degradation. This lysosomal degradation modulates the bioactive substances carried by EVs, thereby shaping the response of the recipient cell.^[^ ^48^, ^49^, ^50^ ^]^ The EVs taken up by recipient cells and processed within lysosomes are capable of influencing cellular signaling pathways.^[^ ^51^ ^]^ Our previous research has shown that EVs activate lysosomal biogenesis in recipient cells via the TFEB‐mediated autophagy pathway.^[^ ^21^, ^22^ ^]^ In the present work, lysosomal redistribution was found to be critical for maintaining intercellular iron balance. CA‐EVs that exhibit lysosomal properties participated in modulating this process. These findings add to the current knowledge of EV relationships with lysosomes.

Ionizing radiation induces ferroptosis, a form of regulated cell death characterized by iron accumulation and lipid peroxidation.^[^ ^14^, ^39^, ^52^, ^53^ ^]^ This process plays a significant role in radiation‐induced bone damage, hindering the regeneration of bone tissues.^[^ ^54^, ^55^, ^56^, ^57^, ^58^ ^]^ Targeting ferroptosis may therefore offer a promising therapeutic approach to improve bone healing and regeneration following ionizing radiation exposure. Current methods using synthesized chemicals or lysosomotropic agents to activate lysosomal iron or induce lysosomal impairments have been developed to sensitize cancers to ferroptosis therapy.^[^ ^11^, ^12^, ^13^ ^]^ In this study, CA‐EVs with lysosomal characteristics were shown to rescue irradiated lysosomal defects, restore recipient iron homeostasis, and modulate endothelial ferroptotic pathways. Furthermore, in vivo results revealed that CA‐EVs not only promote vascular regeneration in irradiated tissues but also specifically mitigate ferroptosis in post‐irradiation mandibular defects. These findings highlight the potential of CA‐EVs as a therapeutic strategy for ferroptosis‐related diseases.

Despite these encouraging results, there are several limitations and challenges associated with the study. First, although lysosomal redistribution mediated by CA‐EVs demonstrated therapeutic potential, the exact mechanisms by which these vesicles interact with host cells and influence lysosomal function post‐irradiation require further investigation. It has been recently revealed that lysosomes can fuse to the plasma membrane and release lysosomal acid sphingomyelinase to trigger irradiation‐induced cell death, which, however, has the controversial role of promoting plasma membrane repair rather than inducing permeabilization.^[^ ^13^, ^37^ ^]^ Thus, the lysosomal behaviors regulated by CA‐EVs would warrant in‐depth observations. Second, the downstream effector(s) of lysosomal regulation modulating the iron flux and mediating ferroptosis control in CA‐EV‐rescued ECs remain to be dissected. Although multiple ferroptosis regulators are involved related to lysosomal regulation, one particular possible factor identified in this study is NCOA4, which responds to irradiation and delivers ferritin to lysosomes via autophagosomes.^[^ ^17^, ^59^, ^60^, ^61^ ^]^ It has been reported that insufficient NCOA4 reduces iron bioavailability and protects cells from free iron participating in the generation of ROS via Fenton‐like reactions.^[^ ^61^, ^62^ ^]^ Examinations at the protein level of NCOA4 and ferritin would be useful for this research. Finally, the complexity and heterogeneity of EV populations pose significant challenges to the standardization of their therapeutic application,^[^ ^63^, ^64^, ^65^, ^66^ ^]^ and standard ferroptosis cells and classical ferroptosis inducers should be adopted to robustly and reproducibly evaluate the anti‐ferroptosis effect of CA‐EVs under essential cell death readout systems.^[^ ^40^ ^]^ Future research should aim to identify the specific molecular components within CA‐EVs responsible for lysosomal modulation and to optimize these vesicles for clinical applications.

## 4 Conclusion

Lysosomal redistribution occurs via HIF‐2α regulation and is mediated by EVs in multi‐stem cell aggregates, which controls iron homeostasis for promoting ECM synthesis and safeguarding cell aggregation. These CA‐EVs exert anti‐ferroptosis effects in recipient irradiated ECs through rescue of lysosomal impairments and restoration of iron homeostasis. CA‐EVs can be harnessed for therapeutic use in promoting CD31^+^EMCN^+^ specialized angiogenesis and promoting bone regeneration of the post‐irradiation mandibular defect.

## 5 Experimental Section

### Mice

All experiments were conducted in accordance with relevant laws and ethical regulations, following the Guidelines of Intramural Animal Use and Care Committees of the Fourth Military Medical University. The study was approved by the Fourth Military Medical University Ethics Committee (protocol number: 2024kq069) and adhered to the Animal Research: Reporting of In Vivo Experiments (ARRIVE) guidelines. A post‐irradiation mandibular defect model was established using male C57BL/6J mice. The mice were obtained from the Laboratory Animal Center of the Fourth Military Medical University. The mice were housed in a specific pathogen‐free environment maintained at 24 °C, with a 12‐h light/dark cycle, 50% humidity, and access to food and water ad libitum. Power analyses based on pilot and published data indicated that using 3 to 4 mice per group provided 80% power with α set to 0.05. Fifty‐six mice (8‐week‐old, 20–25 g) were used to evaluate the function of CAs or CA‐EVs in vivo.

### Cell Lines

The HUVEC line was obtained from the American Type Culture Collection (ATCC, USA; Cat#PCS‐100‐010). The culture and use of HUVECs were approved by the Fourth Military Medical University Ethics Committee (protocol number: IRB‐REV‐2022187). The HUVECs were cultured in EC medium (Sciencell, USA; Cat#1001) at 37 °C with 5% CO_2_. The medium was replaced every three days. The cells were passaged upon reaching 80% confluence.

### Primary Cell Cultures

Stem cells were obtained from clinically available human exfoliated deciduous teeth as previously described,^[^ ^67^ ^]^ under approved protocols by the Fourth Military Medical University Ethics Committee (protocol number: IRB‐REV‐2022187). The cells were cultured in Alpha‐Minimum Essential Medium (α‐MEM; HyClone, USA; Cat#SH30265.01) supplemented with 10% fetal bovine serum (FBS; HyClone, USA; Cat#SH30396.02), 2 mM L‐glutamine (Invitrogen, USA; Cat#A2916801), and 1% penicillin/streptomycin (Invitrogen, USA; Cat#15140122) at 37 °C with 5% CO_2_. Cells at the fifth passage were plated in glass‐bottom dishes (Sorfa, China; Cat#201100), cultured for 24 h, and subsequently used for experiments.

### Induction of CA Formation

Twenty‐four‐well ULA plates (Corning, USA; Cat#3473) were used to induce CA formation. The cells were plated after reaching the fifth passage. A CA was defined as a grouping of at least five cells, as counting more than five cells in a three‐dimensional mass is challenging. Cell aggregation efficiency was calculated using the formula: Cell aggregation efficiency (%) = 1 − log_600_(Number of CAs + Number of unaggregated cells). Initially, there were ≈600 cells per field, and the cell aggregation efficiency was 0. When all the cells in the field aggregated into a single cluster, the cell aggregation efficiency was 1. One CA was divided into ten equal sections, with the center as the origin and the outermost boundary as the endpoint. Cells in the outermost two sections were classified as the outer cells, while cells in the innermost eight sections were classified as the inner cells. The cells in the hypoxic group were incubated in hypoxia workstations (Baker Ruskinn InvivO_2_, England) at 37 °C during CA formation, with a gas mixture of 1% O_2_, 5% CO_2_, and 94% N_2_ to mimic the hypoxic conditions for in vitro culture.

### Chemical Treatments During Cell Aggregation

Bafilomycin A1 (BafA1; MedChemExpress, USA; Cat#HY‐100558), GW4869 (MedChemExpress, USA; Cat#HY‐19363), PT2385 (MedChemExpress, USA; Cat#HY‐12867), and ferrozine (MedChemExpress, USA; Cat#HY‐137805) were dissolved in dimethyl sulfoxide (DMSO; Beyotime, China; Cat#C2041S‐2). The cells were treated with a culture medium containing 200 nm BafA1, 20 µm GW4869, 10 µm PT2385, or 6.5 mm ferrozine.

### RNA Interference

The cells were transfected with siRNA oligonucleotides using Lipofectamine RNAiMAX (Thermo Fisher Scientific, USA; Cat#13778075) at a final concentration of 30 nm. The siRNAs were synthesized by GenePharma, China, and their sequences are listed below. siTFEB: sense, CAUUAUGCGUCUGGACGAUTT; anti‐sense, AUCGUCCAGACGCAUAAUGTT. siEPAS1: sense, GGCGACAUGAUCUUUCUGUTT; anti‐sense, ACAGAAAGAUCAUGUCGCCTT. Stealth siCtrl duplexes, having a comparable GC content, were employed as controls.

### AFM Nanoindentation Test

Nanoindentation test was performed using an Agilent 5500 AFM (Agilent, USA) with silicon probes (resonance frequency: 13 kHz, force constant: 0.2 N/m, sphere diameter: 2.0 µm; Nanosensors, Switzerland). AFM detection was switched to the contact mode, with at least 10 sites for each condyle and 10 measurements for each site, as indicated by the guidelines of the machine. The mean of those over one hundred measures was calculated to represent the value of that sample.

### Collection and Characterization of EVs

The EVs were collected as previously described.^[^ ^36^ ^]^ To prevent contamination by FBS‐derived EVs, medium containing EV‐depleted FBS was prepared by ultracentrifugation at 100 000 g for 18 h. After culturing for 48 h, the supernatant was collected and centrifuged at 800 g for 10 min to remove cells and debris. The resulting supernatant was further centrifuged at 16 000 g for 30 min to pellet the EVs, which were then washed twice with phosphate‐buffered saline (PBS). Protein concentration of the EVs was measured using a BCA protein assay kit (TIANGEN, China; Cat#PA115‐01). TEM was used to analyze the size and morphology of the EVs, while size distribution and the amount of EVs secreted per cell were determined through NTA with a PMX Zetaview (Particle Metrix, Germany). Expression of EV marker proteins was analyzed using Western blots. The CA‐EVs were incubated with 10 µM PT2385 for 30 min prior to NTA and lysosome staining to determine the effect of HIF‐2α inhibition.

### Proteomics

Protein lysates from CA‐EVs and SC‐EVs were prepared and analyzed using LC‐MS/MS on an Orbitrap Exploris 480 mass spectrometer (Thermo Fisher Scientific, USA) equipped with a NanoSpray III ion source. The raw data were processed with the Proteome Discoverer system (version 2.4.1.15; Thermo Fisher Scientific, USA). Protein identification was carried out by searching against the UniProt database, with a false discovery rate threshold set at 0.01 for both peptides and proteins. Protein quantification was performed using the default parameters in MaxQuant (Max Planck Institute of Biochemistry, Germany). Differentially expressed proteins (DEPs; fold change > 1.5, *p* < 0.05) were selected for further functional analysis based on GO and KEGG annotations.

### TEM Analysis

The CAs and unaggregated SCs were fixed overnight at 4 °C in a solution containing 2.5% glutaraldehyde, 2.0% paraformaldehyde (PFA), and 0.1 m sodium cacodylate buffer (all from MilliporeSigma, USA). After washing, the samples were post‐fixed for 1 h at room temperature in 2.0% osmium tetroxide (MilliporeSigma, USA; Cat#75633‐2ML), rinsed in distilled water, and stained *en bloc* with 2% uranyl acetate (Electron Microscopy Sciences, USA; Cat#22400). The specimens were then dehydrated through a graded ethanol series, infiltrated, and embedded in EMbed‐812 resin (Electron Microscopy Sciences, USA; Cat#14120). Thin sections were stained with uranyl acetate and lead citrate (Electron Microscopy Sciences, USA; Cat#22410), and examined using a JEM‐1010 electron microscope (JEOL, Japan) equipped with a digital camera (Hamamatsu Photonics, Japan) and image capture software (AMT Imaging, USA). Cells with intact nuclear envelopes were selected for imaging, focusing on distinguishable lysosomes. For TEM of CA‐EVs, the collected pellets were resuspended in filtered PBS. The diluted CA‐EVs were absorbed onto glow‐discharged 300‐mesh heavy‐duty carbon‐coated formvar copper grids (ProSciTech, Australia) for 5 min, and excess liquid was blotted using filter paper. The grids were washed twice with distilled water and negatively stained with 2.5% uranyl acetate (Electron Microscopy Sciences, USA; Cat#22400). Wide‐field images of multiple vesicles were captured on a JEM‐1200EX electron microscope (JEOL, Japan).

### Lysosome Staining

An AIE‐lysosome probe (Sangon Biotech, China; Cat#E607255) was used to specifically image lysosomes in live cells. The CAs and unaggregated SCs were incubated with 10 µM AIE‐lysosome probe for 30 min at 37 °C. The samples were then washed three times with PBS and incubated with an F‐actin probe conjugated to Alexa Fluor 488 (two drops per mL of medium; Invitrogen, USA; Cat#R37110) for 30 min at 37 °C, if indicated. After another three PBS rinses, the samples were incubated with Hoechst 33342 (10 µg mL^−1^; MedChemExpress, USA; Cat#HY‐15559) at 37 °C for 10 min. After three additional PBS rinses, the samples were immediately examined using a fluorescence microscope. To detect lysosomes in the EVs, the EVs were labeled with PKH67 (Deeyee, China; Cat#DY50232), and incubated with 10 µM AIE‐lysosome probe for 30 min at room temperature. The EVs were washed twice with PBS, and analyzed using a fluorescence microscope.

### Lysosomal pH Sensing Assay

Lysosensor green DND‐189 (Yeasen, China; Cat#40767ES50) was used to measure the pH of lysosomes. The samples were incubated with 1 µm lysosensor green DND‐189 for 30 min at 37 °C. After this, the samples were incubated with Hoechst 33342 (10 µg mL^−1^) at 37 °C for 10 min. The samples were washed three times with PBS, stored in fresh medium, and immediately analyzed using a fluorescence microscope.

### FerroOrange and LysoTracker Green Staining

The samples were incubated with 1 µm FerroOrange (Dojindo, Japan; Cat#F374) in serum‐free medium at 37 °C for 30 min. The cells were subsequently incubated with 75 nm LysoTracker Green (Beyotime, China; Cat#C1047S) at 37 °C for 30 min and then incubated with Hoechst 33342 (10 µg mL^−1^) at 37 °C for 10 min. After washing three times with PBS, the stained cells were immediately examined using a fluorescence microscope.

### EV Uptake Assay In Vitro

The HUVECs were plated in glass‐bottom dishes (Sorfa, China; Cat#201100) to evaluate EV uptake by these cells in vitro. After 24 h, the cells were incubated with 10 µm AIE‐lysosome probe for 30 min at 37 °C. The samples were then incubated with Hoechst 33342 (10 µg mL^−1^) at 37 °C for 10 min. The EVs were labeled with PKH67, and added to the medium at a concentration of 10 µg mL^−1^. To ensure accuracy, the cells were protected from light throughout the experiment. The samples were analyzed using a fluorescence microscope.

### EC Irradiation

HUVECs at passage six were plated in glass‐bottom dishes. Upon reaching 80% confluence, the cells were exposed to 8 Gy X‐rays at a dose rate of 1.35 Gy min^−1^ using a biological irradiator (RS 2000; Rad Source, USA). The EVs were added to the medium after irradiation. The cells were cultured for 24 h. 2,2′‐Bipyridyl (BP; MilliporeSigma, USA; Cat#V900145), a chelator of Fe^2^⁺, was used to evaluate the impact of intracellular Fe^2^⁺ on EC ferroptosis. The chemical was dissolved in DMSO. The irradiated ECs were treated with a culture medium containing 1 mm BP for 24 h.

### Lipid Peroxidation Assay

A lipid peroxidation sensor (C11‐BODIPY 581/591; MedChemExpress, USA; Cat#HY‐D1301) was used to detect ferroptosis. The cells were incubated with 10 µm C11‐BODIPY 581/591 in serum‐free medium at 37 °C for 30 min. After washing three times with PBS, the cells were incubated with Hoechst 33342 (10 µg mL^−1^) at 37 °C for 10 min. The cells were washed three times with PBS. Then, the reduced form of C11‐BODIPY 581/591 (emitting red fluorescence) and its oxidized form (emitting green fluorescence) were both detected by a fluorescence microscope.

### GSH Assay

The intracellular GSH/GSSG ratio was assessed using a GSH and GSSG assay kit (Beyotime, China; Cat#S0053) following the manufacturer's instructions. Briefly, 1 × 10^6^ cells were harvested, treated with a protein removal reagent, and centrifuged at 10,000 g for 10 min at 4 °C. The GSSG and total GSH levels were quantified according to the protocol, with the data being calibrated using standard curves for GSSG and total GSH. The GSH content was determined by subtracting the GSSG amount from the total GSH. The redox state of glutathione was represented by the GSH/GSSG ratio.

### Cell Viability/Cytotoxicity Assay

The HUVECs were incubated with Calcein‐acetoxymethyl ester (Calcein‐AM)/propidium iodide (PI) (Beyotime, China; Cat#C2015M) according to the instructions of the manufacturer. Then the live (emitting green fluorescence) and the dead cells (emitting red fluorescence) were detected by a fluorescence microscope.

### Tube Formation Assay

The HUVECs were exposed to 8 Gy X‐ray at a dose rate of 1.35 Gy min^−1^. After irradiation, the cells were collected using trypsin and seeded at a density of 2 × 10⁵ cells mL^−1^ (50 µL per well) on an µ‐Slide with 15 wells for tube formation assay (ibidi, Germany; Cat#81506) pre‐coated with Matrigel (Shanghai NovaMedical Technology, China; Cat#082703). Serum‐free medium was used during the assay. The CA‐EVs were added to the medium to evaluate their effects on tube formation. To evaluate the effects of V‐ATPase in CA‐EVs, the vesicles were treated with 200 nM BafA1 for 30 min, and washed twice with PBS before being added to the medium. Tube formation was photographed after 5 h.

### CD31^+^EMCN^+^ Vessel Detection

Samples from the tube formation assay (after 5 h of treatment) were washed with PBS, and fixed with 4% PFA for 30 min at room temperature. After another PBS wash, the samples were blocked with the goat serum (Boster Biological Technology, China; Cat#AR0009) for 30 min at room temperature. The cells were then incubated overnight at 4 °C with the following primary antibodies: anti‐CD31 conjugated to Alexa Fluor 488 (R&D Systems, USA; Cat#FAB3628G; diluted 1:100), and anti‐EMCN (Affinity, China; Cat#DF13357; diluted 1:100). After washing three times with PBS, the cells were incubated with an Alexa Fluor 647 donkey anti‐rabbit IgG (Abcam, UK; Cat#ab150075; diluted 1:200) for 2 h at room temperature. The cells were washed three times with PBS and stored in the fluoroshield mounting medium with 4′,6‐diamidino‐2‐phenylindole (DAPI; Abcam, UK; Cat#ab104139) at 4 °C.

### RNA Isolation and qRT‐PCR

Total RNA was isolated from cells and CAs using MIsZOL Reagent (Mishushengwu, China; Cat#MI00617). cDNA synthesis was carried out with PrimeScript RT Master Mix (Takara, Japan; Cat#RR036A). Then, qRT‐PCR was performed using the TB Green Premix Ex Taq II Kit (Takara, Japan; Cat#RR820A) on a CFX96 Real‐Time PCR System (Bio‐Rad, USA). Gene expression levels were normalized to *glyceraldehyde‐3‐phosphate dehydrogenase* (*GAPDH*) as the internal control. The primer sequences used in this study are provided in Table S2 (Supporting Information), and all primers were synthesized by Tsingke Biotech (China).

### Western Blot

Cell and EV lysates were prepared using cell lysis buffer (Beyotime, China; Cat#P0013J) containing a protease inhibitor cocktail (Roche, Switzerland; Cat#11697498001). After protein extraction and quantification with a BCA protein assay kit (TIANGEN, China; Cat#PA115), the proteins were separated by sodium dodecyl sulfate polyacrylamide gel electrophoresis, transferred to polyvinylidene fluoride membranes (Roche, Switzerland; Cat#3010040001), and blocked with 5% bovine serum albumin (BSA; MP Biomedicals, USA; Cat#FC0077) for 2 h at room temperature. The membranes were incubated overnight at 4 °C with the following primary antibodies: anti‐Caveolin‐1 (Santa Cruz Biotechnology, USA; Cat#sc‐53564; diluted 1:1000), anti‐Flotillin‐1 (Proteintech, China; Cat#15571‐1‐AP; diluted 1:1000), anti‐CD9 (Cell Signaling Technology, USA; Cat#13174; diluted 1:1000), anti‐CD63 (Santa Cruz Biotechnology, USA; Cat#sc‐5275; diluted 1:1000), anti‐CD81 (Santa Cruz Biotechnology, USA; Cat#sc‐166029; diluted 1:1000), anti‐Calnexin (Abcam, UK; Cat#ab22595; diluted 1:1000), anti‐TSG101 (Abcam, UK; Cat#ab125011; diluted 1:1000), anti‐Golgin‐84 (Novus Biologicals, USA; Cat#NBP1‐83352; diluted 1:1000), anti‐Mitofilin (Abclonal, China; Cat#A23530; diluted 1:1000), anti‐ATP6V1B1/B2 (Abcam, UK; Cat#ab200839; diluted 1:1000), anti‐LAMP1 (Abcam, UK; Cat#ab208943; diluted 1:1000), and anti‐Cathepsin B (Santa Cruz Biotechnology, USA; Cat#sc‐365558; diluted 1:200). The membranes were washed three times with Tris‐buffered saline‐Tween (TBS‐T; Solarbio, China; Cat#T1081) and incubated with horseradish peroxidase (HRP)‐conjugated secondary antibodies (Cell Signaling Technology, USA; Cat#7074 or Cat#7076; diluted 1:5000) for 1 h at room temperature. After three additional washes with TBS‐T, protein bands were detected using an enhanced chemiluminescence detection kit (4A Biotech, China; Cat#4AW011‐20) and analyzed with a gel imaging system (Tanon, China; Cat#4600).

### Post‐Irradiation Mandibular Defect Modeling

All animal studies were conducted by investigators blinded to treatment groups. Mice were exposed to 10 Gy X‐rays at a dose rate of 1.35 Gy min^−1^ using a Rad Source biological irradiator. Customized lead blocks were used to shield and protect the body of the mice from X‐rays, allowing exposure only to the head. Mandibular defect surgery was performed after irradiation. A defect measuring 1 mm in diameter and 1 mm in depth was created at a fixed area of the mandible using a columnar dental bur under general anesthesia. Surgically treated, non‐irradiated mice served as blank controls. Pluronic F‐127 (PF127; MilliporeSigma, USA; Cat#P2443) was used as a carrier for the implantation of CAs or CA‐EVs in vivo. This is a thermo‐reversible gel that transforms from a liquid to a semi‐solid gel when the temperature rises from 4 °C to 37 °C. The CAs or CA‐EVs were mixed with 10 µL of PF127, and placed into the post‐irradiation defect. To assess the therapeutic effects of EV secretion, the CAs were treated with 20 µm GW4869 for 24 h, washed twice with PBS, mixed with 10 µL of PF127, and placed into the defect. To investigate the therapeutic effects of V‐ATPase activity, the CA‐EVs were treated with 200 nM BafA1 for 30 min, washed twice with PBS, mixed with 10 µL of PF127, and placed into the defect.

### Micro‐CT Analysis

Bone regeneration at the defect sites was assessed four weeks after defect surgery. At the designated time point, mice were euthanized with an overdose of pentobarbital (200 mg kg^−1^), followed by cervical dislocation. The mandibles were isolated and fixed in 4% PFA for 24 h. Each specimen was scanned using micro‐CT systems (Siemens Inveon, Germany, and PerkinElmer, USA) at a setting of 50 kV and 80 µA. Micro‐CT data were reconstructed and analyzed using VGStudio MAX software (version 2023.25.4107; Volume Graphics, Germany). The region of interest was defined as the defect sites. The BV/TV within this area was calculated.

### Fe^2+^ Assay

The Fe^2+^ concentration in defect sites was quantified with the ferrous iron assay kit (Servicebio, China; Cat#G4323) following the manufacturer's instructions. The cellular ferrous iron colorimetric assay kit (Elabscience, China; Cat#E‐BC‐K881‐M) was used to measure the Fe^2+^ concentration in cells.

### Uptake of EVs In Vivo

The mice underwent mandibular defect surgery to assess EV uptake by ECs in vivo. The EVs were labeled with PKH26 (MilliporeSigma, USA; Cat#MIDI26), mixed with 10 µL of PF127, and placed into the defect. The mice were euthanized after 24 h through an overdose of pentobarbital (200 mg kg^−1^) followed by cervical dislocation. The mandibles were isolated and fixed in 4% PFA for 24 h. Decalcification was performed using quick decalcification solution (PhenoVision Bio, China; Cat#PVB‐3002) at 4 °C for 4 days. The decalcified samples were dehydrated overnight in 30% sucrose at 4 °C and embedded in blue frozen section compound (Leica Biosystems, Germany; Cat#3801481). Ten‐micrometer thick sections were blocked with goat serum for 30 min at room temperature. The sections were then incubated overnight at 4 °C with a primary antibody of anti‐CD31 conjugated to Alexa Fluor 488 (R&D Systems, USA; Cat#FAB3628G; diluted 1:100). After thorough washing, the specimens were mounted using the fluoroshield mounting medium with DAPI. Protection from light was maintained throughout the experiment. The specimens were analyzed using a fluorescence microscope.

### Immunofluorescence Staining

The mandibles of the mice were decalcified in 17% ethylenediamine tetraacetic acid (EDTA; Proandy, China; Cat#10218‐1) at 4 °C for 21 days. The samples were dehydrated overnight in 30% sucrose at 4 °C and embedded in blue frozen section compound. Ten‐micrometer thick sections were permeabilized with 0.3% Triton X‐100 (MP Biomedicals, USA; Cat#194854) for 15 min. Specimens were incubated with goat serum for 1 h at room temperature to block non‐specific binding. Primary antibodies were diluted in the blocking solution as follows: anti‐RUNX2 (Santa Cruz Biotechnology, USA; Cat#sc‐390715; diluted 1:200), anti‐CD31 conjugated to Alexa Fluor 488 (R&D Systems, USA; Cat#FAB3628G; diluted 1:100), anti‐EMCN (Santa Cruz Biotechnology, USA; Cat#sc‐65495; diluted 1:100), anti‐4‐HNE (Abclonal, China; Cat#A26085; diluted 1:200), and anti‐GPX4 (Santa Cruz Biotechnology, USA; Cat#sc‐166570; diluted 1:200). Samples were incubated overnight at 4 °C with the primary antibodies. Secondary antibodies, including an Alexa Fluor 647 conjugated donkey anti‐rabbit IgG (Abcam, UK; Cat#ab150075), a Cy3‐conjugated goat anti‐rat IgG (Yeasen, China; Cat#33308ES60), and a CoraLite Plus 594 goat anti‐mouse IgG (Proteintech, China; Cat#RGAM004), were diluted 1:200 in the blocking solution. The samples were incubated with the secondary antibodies at room temperature for 2 h. Unbound secondary antibodies were removed by washing the samples three times with PBS. After thorough washing, the samples were mounted using the fluoroshield mounting medium with DAPI. The CAs and cells were likewise fixed with 4% PFA, permeabilized with 0.3% Triton X‐100, and incubated with the goat serum to block non‐specific binding. Primary antibodies used for the CAs and cells were diluted in the blocking solution as follows: anti‐Col1a1 (Cell Signaling Technology, USA; Cat#72026; diluted 1:200), anti‐fibronectin (MedChemExpress, USA; Cat#HY‐P80493; diluted 1:100), anti‐ATP6V1A (Abcam, UK; Cat#ab199326; diluted 1:200), anti‐connexin 43 (Merck Millipore, USA; Cat#AB1728; diluted 1:200), anti‐ZO‐1 (Bioss, China; Cat#bs‐1329R; diluted 1:200), anti‐integrin αIIb/β3 (Santa Cruz Biotechnology, USA; Cat#sc‐21783; diluted 1:200), anti‐N‐cadherin (MilliporeSigma, USA; Cat#C3865; diluted 1:200), anti‐HIF‐1α (Abclonal, China; Cat#A22041; diluted 1:100), anti‐HIF‐2α (Abclonal, China; Cat#A7553; diluted 1:200), anti‐HIF‐3α (Abclonal, China; Cat#A20020; diluted 1:50), anti‐GPX4 (Abclonal, China; Cat#A1933; diluted 1:50), and anti‐GPX4 (Santa Cruz Biotechnology, USA; Cat#sc‐166570; diluted 1:200). Samples were incubated overnight with the primary antibodies at 4 °C. After incubation with the primary antibodies, samples were washed three times with PBS. Secondary antibodies, including an Alexa Fluor 647 conjugated donkey anti‐rabbit IgG (Abcam, UK; Cat#ab150075) and an iFluor 488 conjugated goat anti‐mouse IgG (Huabio, China; Cat#HA1125), were diluted 1:200 in blocking solution. The samples were incubated with these secondary antibodies at room temperature for 2 h. Unbound secondary antibodies were removed by washing the samples three times with PBS. After thorough washing, the samples were mounted using the fluoroshield mounting medium with DAPI.

### Image Acquisition and Analyses

Bright‐field images of CA morphology and tube formation were captured using an inverted microscope (Leica, Germany). Fluorescence imaging was performed with a CLSM (A1+; Nikon, Japan). Representative high‐magnified images were shown to assess the cellular details, and low‐magnified image examples were also attached (Figure S7, Supporting Information). As usually one CA was formed in each 24‐well in ULA plates, quantifications were performed by analyzing the number of CAs per condition as the sample *n* indicated. For SC and ECs, quantifications were performed by analyzing the number of high magnified regions per condition as the sample *n* indicated. Image quantification was conducted using ImageJ software (version 1.54g; National Institutes of Health, USA) on distinct samples. For the tube formation assay, the number of network structures was quantified by analyzing three randomly selected microscopic fields using ImageJ software with the “angiogenesis analyzer” plugin.

### Statistical Analysis

Data are expressed as mean ± standard deviation (SD) unless otherwise specified for *n* ≥ 3 without pre‐processing of the data. Statistical significance was assessed using a two‐tailed unpaired Student's *t*‐test for comparisons between two groups. For comparisons among multiple groups, one‐way analysis of variance (ANOVA) with Tukey's post hoc test was used. Cell aggregation efficiency was analyzed with two‐way ANOVA. A value of *p* < 0.05 was considered statistically significant. Statistical analysis and graphing were performed with GraphPad Prism (version 9.0.0; GraphPad Software, USA).

## Conflict of Interest

The authors declare no conflict of interest.

## Author Contributions

Y.Y.L. and B.M. contributed equally to this work. The study was conceptualized by Y.Y.L. and B.D.S. Methodology was developed by Y.Y.L., S.F.B., and B.D.S. All authors participated in the investigation. Visualization was carried out by Y.Y.L. and J.W.L. Funding was acquired by W.Z.W., C.X.Z., X.R.X., J.C., F.J., H.K.X., Y.S., and B.D.S. Project administration was managed by Y.J., Y.S., and B.D.S., who also provided supervision. The original draft was written by Y.Y.L. and B.D.S., with all authors contributing to the review and editing of the manuscript.

## Supporting information



Supporting Information

## Data Availability

All data included in this study are available upon reasonable request of corresponding authors. The raw sequence data reported in this research have been uploaded as a supplement.
