# Supplementary material for: Lysosome‐Featured Cell Aggregate‐Released Extracellular Vesicles Regulate Iron Homeostasis and Alleviate Post‐Irradiation Endothelial Ferroptosis for Mandibular Regeneration
Source: Adv Sci (Weinh). 2025 Jun 23;12(34):e05070. doi: 10.1002/advs.202505070 (PMC12442703; doi:10.1002/advs.202505070)
Supplement: Supplementary file 1 — Supporting Information [file ADVS-12-e05070-s001.docx]

Supporting Information

Lysosome-Featured Cell Aggregate-Released Extracellular Vesicles Regulate Iron Homeostasis and Alleviate Post-Irradiation Endothelial Ferroptosis for Mandibular Regeneration

Yuan-Yuan Li, Bo Ma, Jia-Wei Lu, Kai-Chao Zhang, Chao Ma, Sheng-Feng Bai, Yan-Jiao Li, Si-Qi Ying, Wei-Zong Weng, Kai Zhang, Xi-Wang Hu, Rang Li, Chen-Xi Zheng, Xiao-Ru Xu, Ji Chen, Fang Jin, Hao-Kun Xu, Jian-Wei Xie, Yan Jin, Yi Shuai*, Bing-Dong Sui*


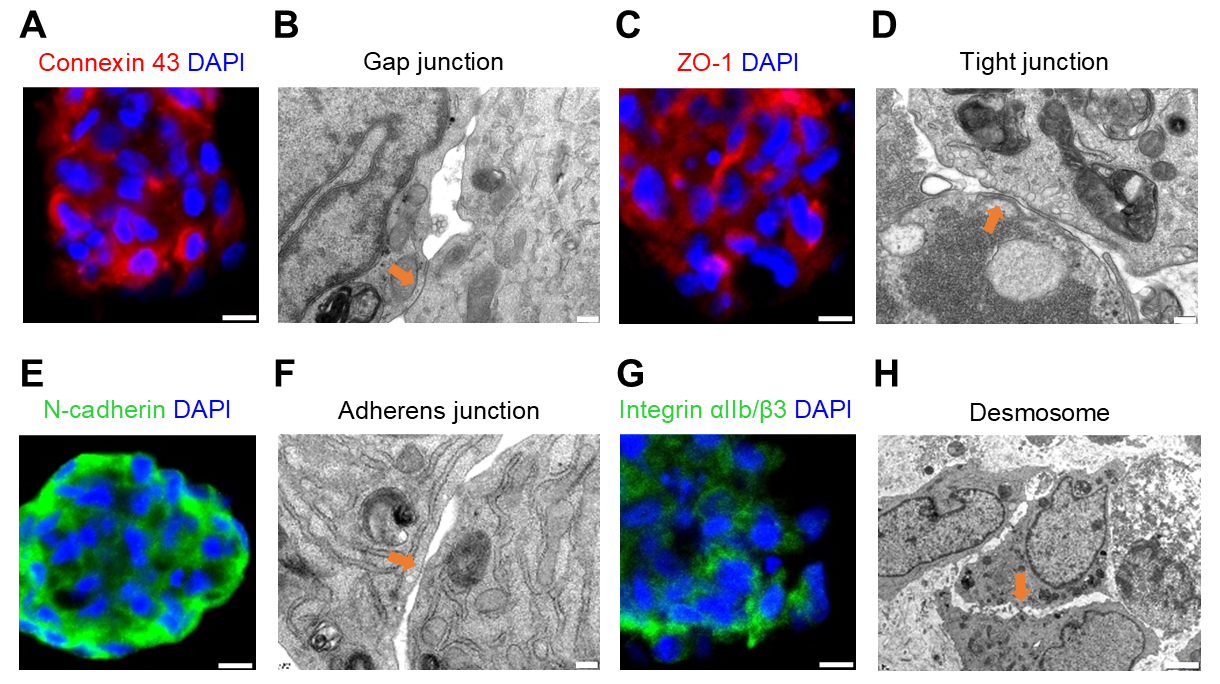


**Figure S1.** Immunostaining and TEM observations of CAs.

(A,C,E,G) Immunostaining for Connexin 43 (red), ZO-1 (red), N-cadherin (green), and Integrin αIIb/β3 (green). Nuclei are stained with DAPI (blue). Scale bars, 10 μm. (B,D,F,H) TEM observations for connections between cells (indicated by arrows). Scale bars are 2 μm for desmosome and 200 nm for others.


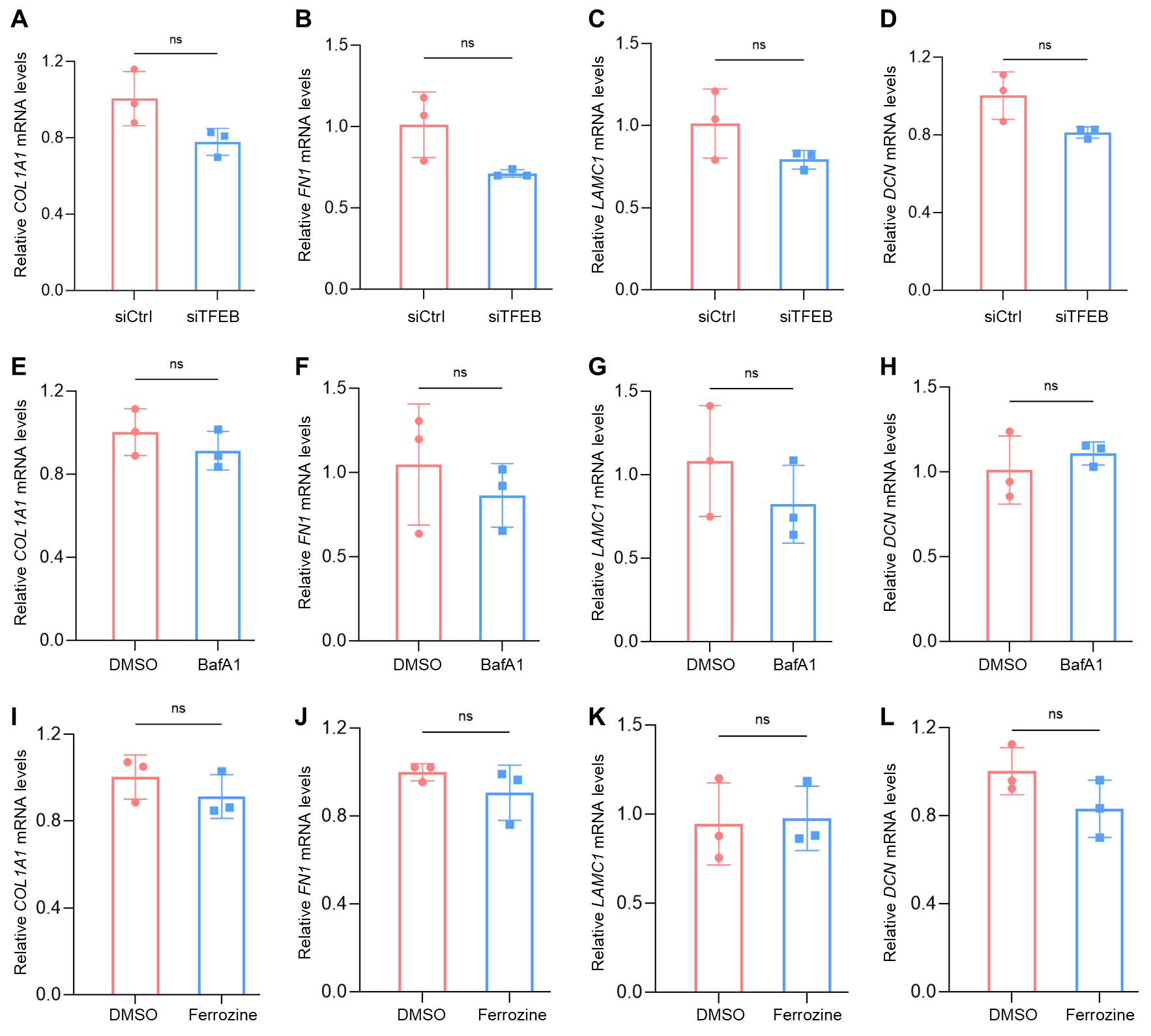


**Figure S2.** The effects of siTFEB*,* BafA1, and ferrozine on ECM gene expression in CAs.

(A,E,I) Gene expression of *COL1A1* in CAs was determined by qRT-PCR. (B,F,J) Gene expression of *FN1* in CAs was determined by qRT-PCR. (C,G,K) Gene expression of *LAMC1* in CAs was determined by qRT-PCR. (D,H,L) Gene expression of *DCN* in CAs was determined by qRT-PCR. siCtrl, siRNA negative control; siTFEB, siRNA oligonucleotides of *transcription factor EB*; DMSO, dimethyl sulfoxide; BafA1, bafilomycin A1. Results are expressed as mean ± SD. *n* = 3 samples per group for each experimental readout. *p* values were calculated using Student's *t*-test. ns, not significant (*p* > 0.05).


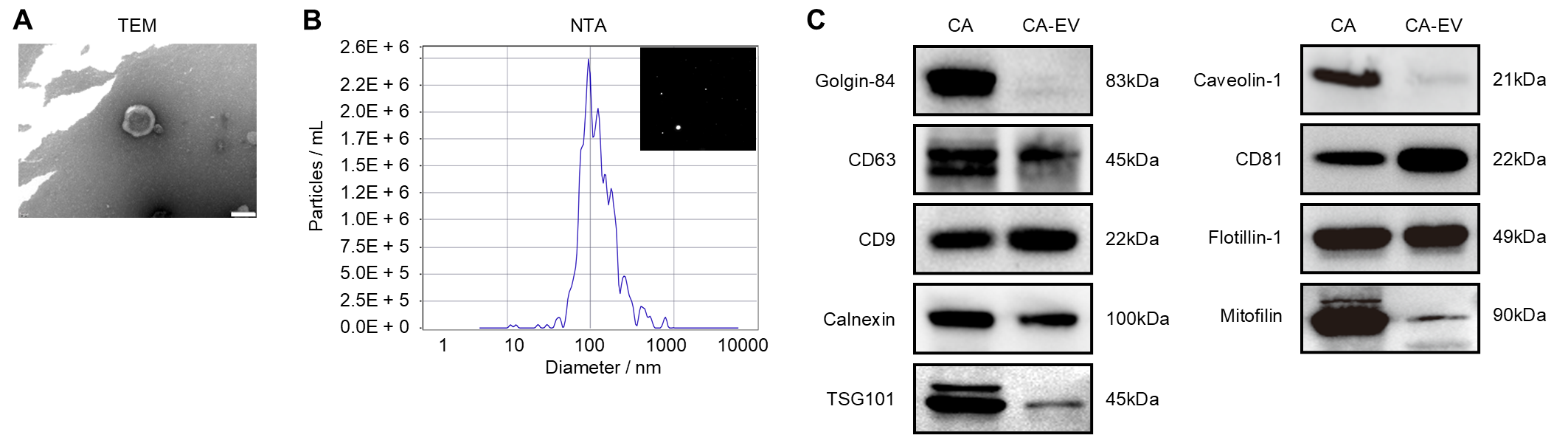


**Figure S3.** Characterization of CA-EVs.

(A) TEM observation for a CA-EV. Scale bar, 200 nm. (B) NTA image of CA-EVs. (C) Protein expression in CA-EVs compared to CAs.


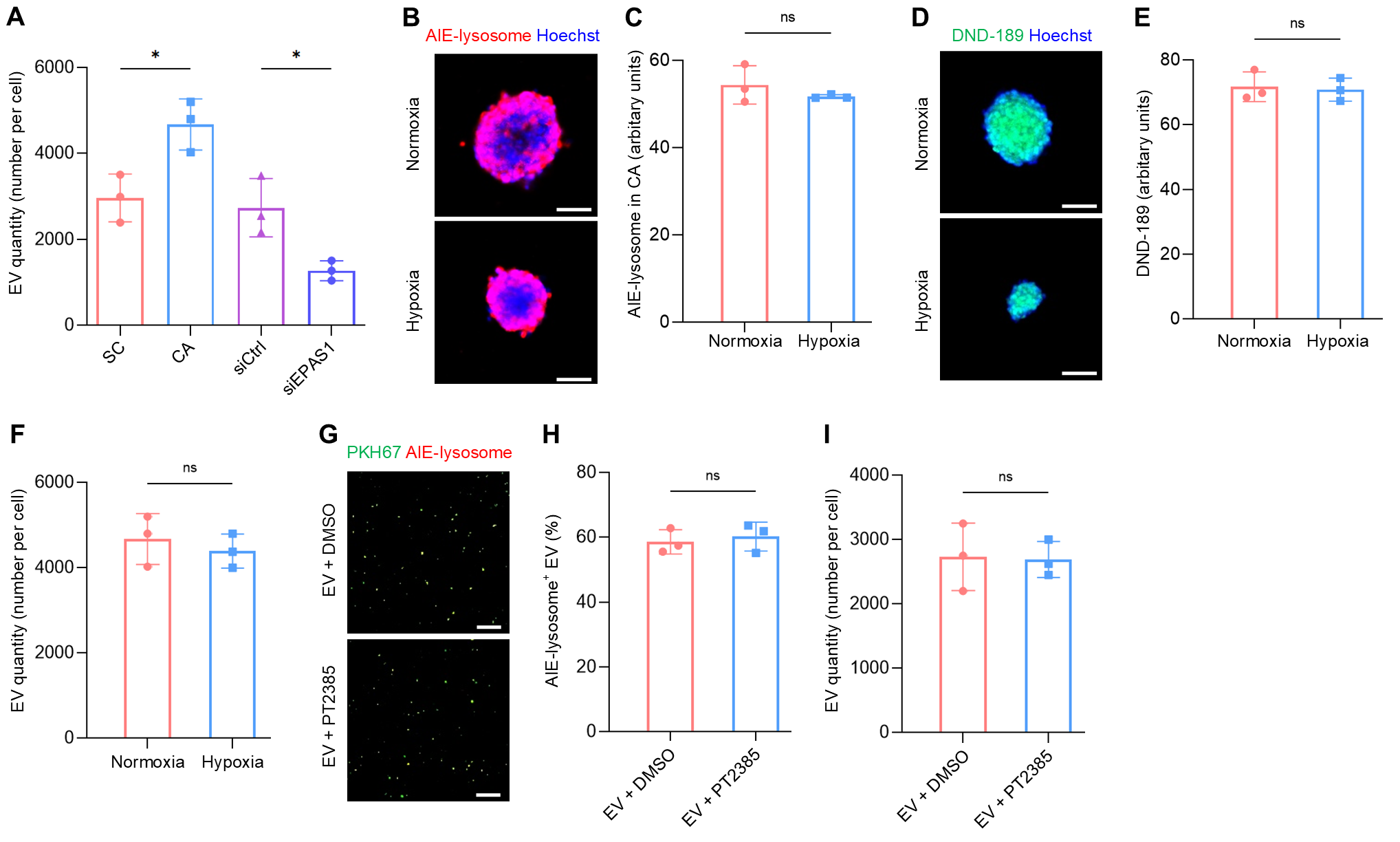


**Figure S4.** Hypoxic regulation of CAs and CA-EVs.

(A) Quantification of EV secretion by NTA. SC, unaggregated stem cell; CA, cell aggregate; siCtrl, siRNA negative control treating CA; siEPAS1, siRNA oligonucleotides of *endothelial PAS domain-containing protein 1* (*EPAS1*, encoding HIF-2α) treating CA. (B) Fluorescent staining of AIE-lysosome (red) in CAs, with nuclei stained with Hoechst (blue). Scale bars, 50 μm. (C) Quantification of AIE-lysosome fluorescent intensity in (B). (D) Fluorescent staining of the pH indicator, lysosensor DND-189 (green), in CAs, with nuclei stained with Hoechst (blue). Scale bars, 50 μm. (E) Quantification of DND-189 fluorescent intensity in (D). (F) Quantification of EV secretion from CAs by NTA. Hypoxia, 1% O_2_ during culture of CAs. (G) Fluorescent staining of AIE-lysosome (red) co-stained with PKH67 (green) in CA-EVs. Scale bars, 10 μm. (H) Quantification of the proportion of AIE-lysosome^+^ EVs in (G). (I) Quantification of CA-EVs by NTA. PT2385, a HIF-2α inhibitor treating CA-EVs. Results are expressed as mean ± SD. *n* = 3 samples per group for each experimental readout. *p* values were calculated using Student's *t*-test (C,E,F,H,I), and one-way ANOVA with Tukey's post hoc test (A). **p* < 0.05; ns, not significant (*p* > 0.05).


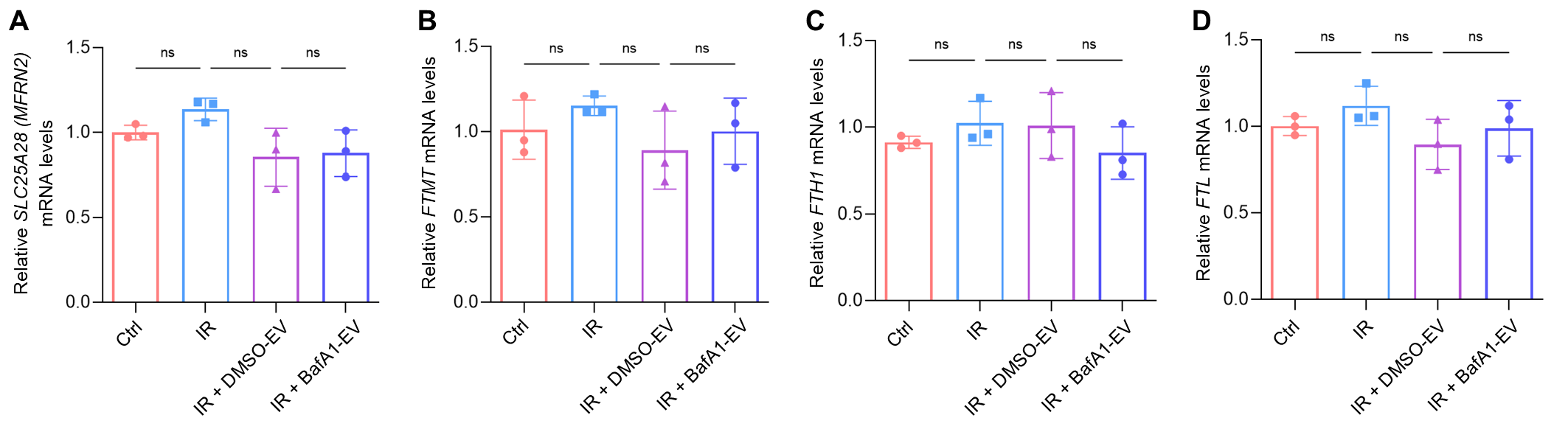


**Figure S5.** Gene expression in HUVECs.

(A-D) Gene expression levels of *SLC25A28* (encoding *MFRN2*), *FTMT*, *FTH1*, and *FTL* in HUVECs was determined by qRT-PCR. Ctrl, control; IR, irradiation; DMSO-EV, EV secreted by CA treated with dimethyl sulfoxide; BafA1-EV, EV secreted by CA treated with bafilomycin A1. Results are expressed as mean ± SD. *n* = 3 samples per group for each experimental readout. *p* values were calculated using one-way ANOVA with Tukey's post hoc test. ns, not significant (*p* > 0.05).


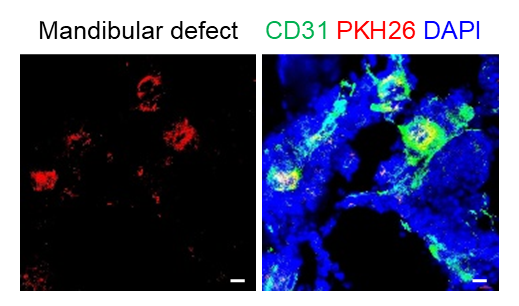


**Figure S6.** Uptake of CA-EVs *in vivo*.

Fluorescent staining of PKH26 (red) co-immunostained with CD31 (green) in the mandibular defect region. Nuclei are stained with DAPI (blue). Scale bars, 10 μm.


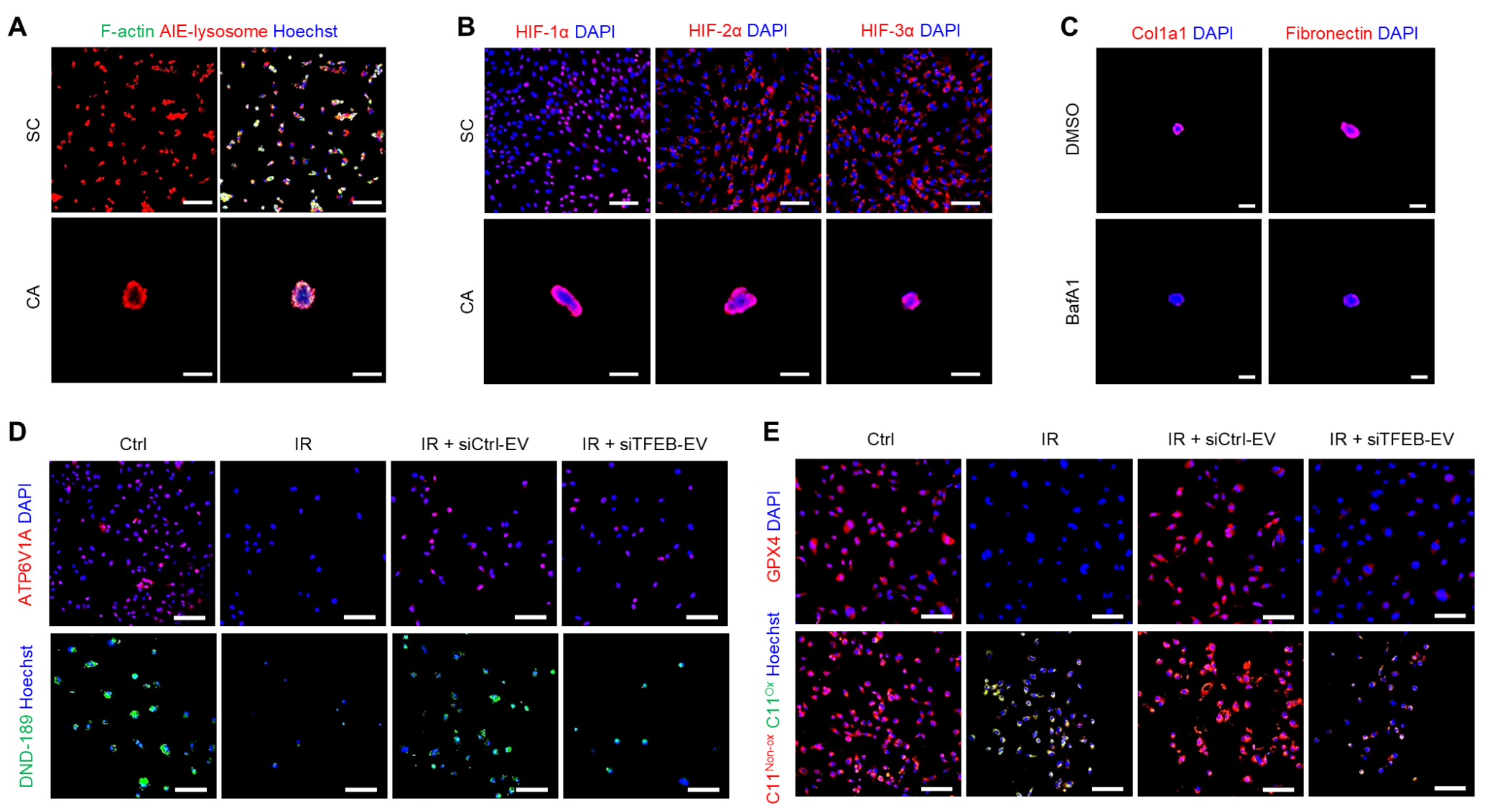


**Figure S7.** Low-magnified image examples of fluorescent staining.

(A) Fluorescent staining of AIE-lysosome (red) co-stained with F-actin (green), with nuclei stained with Hoechst (blue). Scale bars, 100 μm. (B) Immunostaining for HIF-1α, HIF-2α, and HIF-3α (red), with nuclei stained with DAPI (blue). Scale bars, 100 μm. SC, unaggregated stem cell; CA, cell aggregate. (C) Immunostaining for Col1a1 or fibronectin (red) in CAs, with nuclei stained with DAPI (blue). Scale bars, 100 μm. DMSO, dimethyl sulfoxide; BafA1, bafilomycin A1. (D) Immunostaining for ATP6V1A (red) with nuclei stained with DAPI (blue), or fluorescent staining of DND-189 (green) with nuclei stained with Hoechst (blue) in the HUVECs. Scale bars, 100 μm. (E) Immunostaining for GPX4 (red) with nuclei stained with DAPI (blue), or fluorescent staining of C11-BODIPY (red, non-oxidized; green, oxidized) in HUVECs with nuclei stained with Hoechst (blue). Scale bars, 100 μm. Ctrl, control; IR, irradiation; siCtrl-EV, EV secreted by CA treated with siRNA negative control; siTFEB-EV, EV secreted by CA treated with siRNA oligonucleotides of *Transcription Factor EB*.

**Table S1.** List of all the proteins identified during proteomic analysis.

See attachment.

**Table S2.** Primer sequences for qRT-PCR.

| Primer name | Primer sequences (5’-3’) |
| --- | --- |
| *h-COL1A1-F* | GAGGGCCAAGACGAAGACATC |
| *h-COL1A1-R* | CAGATCACGTCATCGCACAAC |
| *h-FN1-F* | CGGTGGCTGTCAGTCAAAG |
| *h-FN1-R* | AAACCTCGGCTTCCTCCATAA |
| *h-LAMC1-F* | GGACTCCGCCCGAGGAATA |
| *h-LAMC1-R* | ACTTGAGACGCACATAGGTGA |
| *h-DCN-F* | ATGAAGGCCACTATCATCCTCC |
| *h-DCN-R* | GTCGCGGTCATCAGGAACTT |
| *h-PTGS2-F* | TAAGTGCGATTGTACCCGGAC |
| *h-PTGS2-R* | TTTGTAGCCATAGTCAGCATTGT |
| *h-AIFM2-F* | AGACAGGGTTCGCCAAAAAGA |
| *h-AIFM2-R* | CAGGTCTATCCCCACTACTAGC |
| *h-DHODH-F* | CCACGGGAGATGAGCGTTTC |
| *h-DHODH-R* | CAGGGAGGTGAAGCGAACA |
| *h-SLC7A11-F* | TCTCCAAAGGAGGTTACCTGC |
| *h-SLC7A11-R* | AGACTCCCCTCAGTAAAGTGAC |
| *h-GAPDH-F* | GCACCGTCAAGGCTGAGAAC |
| *h-GAPDH-R* | TGGTGAAGACGCCAGTGGA |
| *h-GPX4-F* | GAGGCAAGACCGAAGTAAACTAC |
| *h-GPX4-R* | CCGAACTGGTTACACGGGAA |
| *h-ACSL4-F* | CATCCCTGGAGCAGATACTCT |
| *h-ACSL4-R* | TCACTTAGGATTTCCCTGGTCC |
| *h-STEAP3-F* | CTCCCCGGAGGTCATCTTTG |
| *h-STEAP3-R* | TCTTGCTCTGTAGGGTTGCTC |
| *h-DMT1-F* | TGGAGATCATGGGGAGTCTG |
| *h-DMT1-R* | AAGAAAACCTGGTCCGGTGAA |
| *h-SLC40A1-F* | CTACTTGGGGAGATCGGATGT |
| *h-SLC40A1-R* | CTGGGCCACTTTAAGTCTAGC |
| *h-MFRN2-F* | GTACCCCATCGACTGCGTC |
| *h-MFRN2-R* | CTCCAACACATTGCGATAGCG |
| *h-FTMT-F* | TGGAGTGTGCTCTACTCTTGG |
| *h-FTMT-R* | ACGTGGTCACCTAGTTCTTTGA |
| *h-NCOA4-F* | GAGGTGTAGTGATGCACGGAG |
| *h-NCOA4-R* | GACGGCTTATGCAACTGTGAA |
| *h-FTH1-F* | CCCCCATTTGTGTGACTTCAT |
| *h-FTH1-R* | GCCCGAGGCTTAGCTTTCATT |
| *h-FTL-F* | CAGCCTGGTCAATTTGTACCT |
| *h-FTL-R* | GCCAATTCGCGGAAGAAGTG |
| *h-TFR1-F* | ACCATTGTCATATACCCGGTTCA |
| *h-TFR1-R* | CAATAGCCCAAGTAGCCAATCAT |
| *h-EPAS1-F* | CGGAGGTGTTCTATGAGCTGG |
| *h-EPAS1-R* | AGCTTGTGTGTTCGCAGGAA |
| *h-TFEB-F* | ACCTGTCCGAGACCTATGGG |
| *h-TFEB-R* | CGTCCAGACGCATAATGTTGTC |
